# Lymph-Node Resident CD8α^+^ Dendritic Cells Capture Antigens from Migratory Malaria Sporozoites and Induce CD8^+^ T Cell Responses

**DOI:** 10.1371/journal.ppat.1004637

**Published:** 2015-02-06

**Authors:** Andrea J. Radtke, Wolfgang Kastenmüller, Diego A. Espinosa, Michael Y. Gerner, Sze-Wah Tse, Photini Sinnis, Ronald N. Germain, Fidel P. Zavala, Ian A. Cockburn

**Affiliations:** 1 Johns Hopkins Malaria Research Institute and Department of Molecular Microbiology and Immunology, Johns Hopkins Bloomberg School of Public Health, Baltimore, Maryland, United States of America; 2 Lymphocyte Biology Section, Laboratory of Systems Biology, National Institute of Allergy and Infectious Diseases, National Institutes of Health, Bethesda, Maryland, United States of America; National Institute for Medical Research, UNITED KINGDOM

## Abstract

Malaria infection begins when a female *Anopheles* mosquito injects *Plasmodium* sporozoites into the skin of its host during blood feeding. Skin-deposited sporozoites may enter the bloodstream and infect the liver, reside and develop in the skin, or migrate to the draining lymph nodes (DLNs). Importantly, the DLN is where protective CD8^+^ T cell responses against malaria liver stages are induced after a dermal route of infection. However, the significance of parasites in the skin and DLN to CD8^+^ T cell activation is largely unknown. In this study, we used genetically modified parasites, as well as antibody-mediated immobilization of sporozoites, to determine that active sporozoite migration to the DLNs is required for robust CD8^+^ T cell responses. Through dynamic *in vivo* and static imaging, we show the direct uptake of parasites by lymph-node resident DCs followed by CD8^+^ T cell-DC cluster formation, a surrogate for antigen presentation, in the DLNs. A few hours after sporozoite arrival to the DLNs, CD8^+^ T cells are primed by resident CD8α^+^ DCs with no apparent role for skin-derived DCs. Together, these results establish a critical role for lymph node resident CD8α^+^ DCs in CD8^+^ T cell priming to sporozoite antigens while emphasizing a requirement for motile sporozoites in the induction of CD8^+^ T cell-mediated immunity.

## Author Summary

Malaria is responsible for the deaths of 0.5–2 million people each year. A safe and effective vaccine is likely needed for the control or eradication of malaria. Immunization with irradiated sporozoites, the infectious stage of the parasite transmitted by mosquitoes, protects people against malaria through the activation of specialized effector cells called CD8^+^ T cells, which can eliminate live parasites. The induction of such malaria-specific CD8^+^ T cells is critically dependent on dendritic cells, a diverse population of antigen-presenting cells. It was previously unclear how dendritic cells acquire sporozoite antigens to induce the protective CD8^+^ T cell response. Using a combination of functional studies and high-resolution imaging, we report here that live sporozoites access skin-draining lymph nodes after infection and directly provide antigens to resident dendritic cells that in turn activate CD8^+^ T cells. These results underscore the importance of live, motile sporozoites in the induction of protective CD8^+^ T cell responses and provide a mechanistic understanding for the superior immunogenicity of whole parasite vaccines.

## Introduction

Sterile immunity against live sporozoite challenge is elicited by immunization with radiation-attenuated sporozoites [1] and is, in part, mediated by CD8^+^ T cells specific for the *Plasmodium* circumsporozoite (CS) antigen [2, 3]. Using a model mimicking natural exposure to sporozoite-infected mosquitoes, we previously demonstrated that CS-specific CD8^+^ T cell responses are primed by DCs in the skin-draining lymph nodes (DLNs) of mice [4]. Following activation in the DLNs, CS-specific CD8^+^ T cells migrate to the liver where they eliminate parasite-infected hepatocytes [4, 5]. Subsequently, others have shown that immune responses generated in the DLNs are sufficient for sterile protection against live sporozoites [6]. These findings challenged the prevalent idea that CD8^+^ T cell responses against malaria liver stages originate exclusively in hepatic tissues.

How do skin-deposited sporozoites elicit cell-mediated immune responses in the DLNs? The induction of malaria-specific CD8^+^ T cells is critically dependent on dendritic cells (DCs) [4, 7–11], a diverse population of specialized antigen-presenting cells (APCs). The phenotypic diversity of DCs is exemplified in murine skin-DLNs which contain lymphoid-tissue resident DCs (composed of CD8α^+^ and CD11b^+^ subsets), B220^+^ plasmacytoid DCs, and three distinct subsets of skin-derived migratory DCs [12]. In addition, DCs differ in their ability to present antigen to CD4^+^ and CD8^+^ T cells [12, 13] and are located within different compartments in the DLN [14, 15]. For any cutaneously-deposited pathogen or vaccine, this phenotypic and spatial heterogeneity raises the question of how antigen is transported to the secondary lymphoid tissue and which DCs are responsible for priming CD8^+^ T cells. This issue is especially important for malaria given that immunization with sporozoites represents the gold-standard for malaria vaccination and understanding the factors that contribute to efficient antigen presentation may aid vaccine design [16].

Several studies have examined T cell, APC, and parasite interactions in infections other than malaria [17,18]; however, the role of different DC subsets in the transport and presentation of parasite antigens is not well understood or, in the case of *Leishmania* infection, is controversial [19, 20]. In contrast, these questions have been well studied in viral models. In infections with tissue-tropic viruses, such as influenza virus and *Herpes simplex* virus (HSV), tissue-derived DCs play prominent roles in either the transport of antigen to lymph node (LN)-resident DCs or the direct presentation of antigen to CD8^+^ T cells [21, 22]. In other infections such as vaccinia virus in which the virus can infect dendritic cells, direct presentation of antigen to CD8^+^ T cells has been observed just beneath the subcapsular sinus [23] and within the LN parenchyma [24, 25].

Based on these viral paradigms, there are several potential routes by which antigen might be presented to malaria-specific CD8^+^ T cells after skin delivery of sporozoites. One possibility is that sporozoite antigen is acquired in the dermis by skin-resident migratory DCs, trafficked to the DLNs, and presented directly to CD8^+^ T cells. Alternatively, skin-emigrant DCs may transfer antigens to LN-resident DCs for presentation and CTL activation. These models are supported by the fact that sporozoites are injected and, in some cases, develop in the skin after mosquito inoculation (reviewed in [26]). However, these models fail to take into account the sporozoites’ exquisite motility and the superior immunogenicity of live, irradiated sporozoites versus dead sporozoites. Therefore, we investigated a third possibility: that CD8^+^ T cell priming does not require skin-derived DCs, but instead, depends on antigen delivery to lymphoid tissues by migratory parasites. To acquire insight into these issues, we used genetically manipulated parasites, advanced imaging technologies, and transgenic mice with constitutive or conditional loss of distinct APC subsets. We found that skin-derived DCs are not needed for CD8^+^ T cell priming; rather the ability of parasites to actively traverse out of the skin is critical for the induction of CD8^+^ T cell responses by LN-resident DCs. Our data provide the most complete picture to date of the events required for the development of an adaptive cell-mediated immune response against skin-deposited malaria liver stages.

## Results

### Langerhans cells and langerin^+^ dermal DCs are not required for priming CS-specific CD8^+^ T cells in the DLN

**Figure 1:**
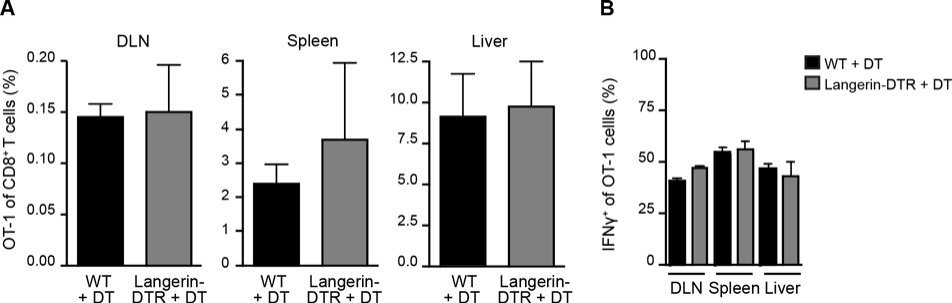
Langerhans cells and langerin^+^ dermal DCs are dispensable for anti-CS CD8^+^ T cell responses. MuLangerin-DTR/EGFP and C57BL/6 mice received a single IP injection of DT (1 μg). 24 hours after DT treatment, 5×10^3^ naive OT-1 cells were adoptively transferred to recipient mice. Mice were immunized through the bites of 20 irradiated *P*. *berghei* CS^5M^-infected mosquitoes 1 day after cell transfer and 2 days after DT treatment. A. Percentage of OT-1 cells of total CD8^+^ T cells recovered 10 days after sporozoite inoculation. B. Percentage of OT-1 cells producing IFN-γ as determined by intracellular staining and flow cytometry 10 days after inoculation by irradiated *P*. *berghei* CS^5M^–infected mosquito bites (mean ± SEM; n = 4–5/group). Data representative of 2 similar experiments.

Given the prolonged residence and development of parasites in the skin after inoculation [27, 28], we hypothesized that migratory skin DCs may be critically involved in CD8^+^ T cell priming, either by direct presentation of sporozoite antigens or via transfer of such antigens to LN-resident DCs. To evaluate the contribution of skin-derived migratory DCs we used a knock-in mouse model in which the diphtheria toxin receptor (DTR) is expressed under the control of the murine langerin promoter [15]. Administration of diphtheria toxin (DT) to these animals rapidly depletes Langerhans cells in the epidermis (which only recover after ~2 weeks), langerin^+^ CD103^+^ DCs in the dermis (which begin to recover after ~ 5 days), and langerin-expressing skin-emigrant DCs in the DLN [15, 29–31] and S1A-C Fig. Though langerin is expressed by a population of LN-resident CD8α^+^ DCs [15, 30], this subset is missing on the C57BL/6 background [32]. Therefore, MuLangerin-DTR/EGFP mice on the C57BL/6 background allow us to examine the role of Langerhans cells and langerin^+^ dermal DCs in CD8^+^ T cell priming without affecting LN-resident CD8α^+^ DCs. Following depletion of these subsets with DT, we transferred OT-1 TCR transgenic T cells specific for the H-2K^b^-SIINFEKL ligand into treated animals and immunized these mice with *P*. *berghei* CS^5M^ sporozoites, a transgenic parasite expressing SIINFEKL within the CS protein [7]. To mimic the natural situation as closely as possible, mice were immunized through the bites of *P*. *berghei* CS^5M^-infected mosquitoes and the magnitude of the OT-1 response was examined in the DLNs, spleens, and livers 10 days later. There was no difference in the expansion nor antigen-specific IFN-γ production of OT-1 cells recovered from WT and MuLangerin-DTR/EGFP mice treated with DT (Fig. 1 and S1D Fig.). These data demonstrate that Langerhans cells and langerin^+^ dermal DCs are dispensable for the transport and/or presentation of sporozoite antigens to CD8^+^ T cells in the DLN.

### Sporozoite migration to the DLN is required for CD8^+^ T cell priming

**Figure 2:**
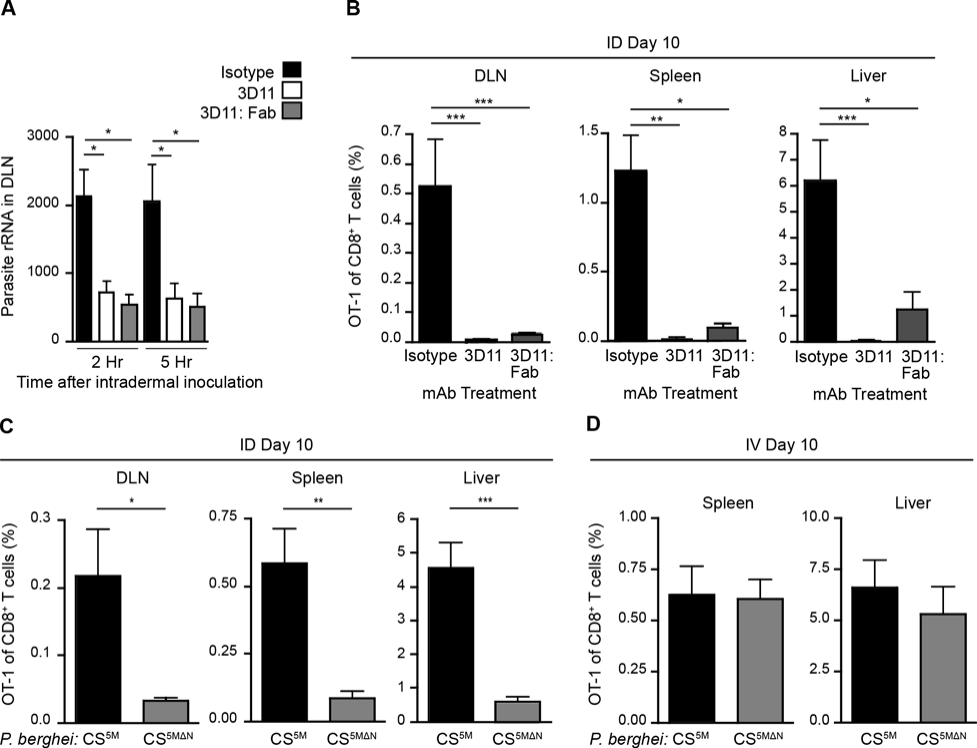
Sporozoite migration to the DLN is required for robust CD8^+^ T cell priming. A. Naïve mice were injected ID with 2×10^4^ *P*. *berghei* CS^5M^ sporozoites treated for 20 minutes with 25 ug/ml of mAb, 2G3 (isotype control) or anti-*P*. *berghei* CS (3D11), or 16 ug/ml of Fab fragments prepared from the 3D11 antibody. Total RNA was isolated from DLNs at the indicated times. Parasite burdens were quantified by RT-PCR and pooled from 2 similar experiments; mean ± SEM, n = 10/group. B. 5×10^3^ naïve OT-1 cells were transferred to mice. 24 hours later, mice were injected ID with 2×10^4^ irradiated *P*. *berghei* CS^5M^ sporozoites treated with 25 ug/ml 2G3 or 3D11 mAb or 16 ug/ml 3D11:Fab. Proportion of CD8^+^ T cells of OT-1 origin recovered 10 days post-inoculation; n = 5/group, mean ± SEM. Data representative of 2 similar experiments. C. 5×10^3^ naïve OT-1 cells were transferred to mice. Mice were injected ID with 2×10^4^ irradiated *P*. *berghei* CS^5M^ or *P*. *berghei* CS^5MΔN^ sporozoites 1 day after cell transfer. Proportion of CD8^+^ T cells of OT-1 origin recovered 10 days post-inoculation; n = 5/group, mean ± SEM. Data are representative of 4 similar experiments. D. 5×10^3^ naive OT-1 cells were transferred to mice. 1 day after transfer mice were immunized IV with 2×10^4^ irradiated *P*. *berghei* CS^5M^ sporozoites or *P*. *berghei* CS^5MΔN^ sporozoites. Expansion of OT-1 cells was measured 10 days after inoculation; mean ± SEM, n = 4–6/group. Data representative of 3 similar experiments.

Having established a nonessential role for Langerhans cells and langerin^+^ dermal DCs in sporozoite antigen presentation to CD8^+^ T cells, we hypothesized that the parasites themselves may deliver antigens to LN-resident DCs. To investigate the contribution of direct parasite access to the DLN for T cell activation, we applied two approaches. First, we limited parasite motility by antibody-mediated immobilization [33–35]. To this end, we pre-treated sporozoites with a CS-specific mAb before intradermal (ID) injection of the parasites. Additionally, we used monovalent Fab fragments in these experiments to avoid potential opsonization of the organism. We then assessed the parasite burden in the DLNs at 2 and 5 hours post-inoculation, as this is when we have previously detected the highest numbers of sporozoites in this organ [4]. Strikingly, both full-length CS-specific mAb (3D11) and monovalent Fab fragments (3D11:Fab) significantly reduced parasite burden in the DLN as compared to parasites incubated with a control mAb (Fig. 2A). Such a reduction in direct parasite access to the DLN was accompanied by a dramatic reduction in the clonal expansion of antigen-specific CD8^+^ T cells in the DLN as well as the spleen and liver (Fig. 2B), two sites of effector T cell migration after initial priming in the DLN [4].

As a second approach, we generated mutant parasites that expressed the SIINFEKL epitope in a CS-restricted manner and were unable to migrate out of the skin due to a deletion in the N-terminal third of the CS protein [36], *P*. *berghei* CS^5MΔN^ (S2A-C Fig.). In further agreement with the hypothesis that sporozoite motility is required for parasite entry into the lymphatics, *P*. *berghei* CS^5MΔN^ sporozoites exhibited a severe impairment in their migration to the DLN (S2D Fig.). To evaluate the effect of these changes in DLN access on the development of cell-mediated immunity, mice were given CFSE-labeled OT-1 cells, injected ID with *P*. *berghei* CS^5M^ or *P*. *berghei* CS^5MΔN^ sporozoites, and OT-1 proliferation was examined 3 days later in the DLN. OT-1 proliferation was significantly reduced in mice injected with *P*. *berghei* CS^5MΔN^ sporozoites (S2E and F Fig.). To determine whether the diminished CD8^+^ T cell response observed at day 3 was due to a delay in sporozoite migration to the DLN, rather than an absolute reduction, we measured the expansion of OT-1 cells in the DLN, spleen, and liver 10 days after ID inoculation of *P*. *berghei* CS^5M^ or *P*. *berghei* CS^5MΔN^ sporozoites. In line with our previous findings, we found that ID immunization with *P*. *berghei* CS^5MΔN^ sporozoites led to drastically reduced OT-1 responses 10 days after immunization as compared to *P*. *berghei* CS^5M^ sporozoites (Fig. 2C). Importantly, when injected IV, *P*. *berghei* CS^5MΔN^ sporozoites were fully infectious (S2G Fig.) and induced similar OT-1 responses in the spleen and liver as compared to control parasites (*P*. *berghei* CS^5M^) (Fig. 2D). These findings demonstrate that the SIINFEKL epitope is expressed by the mutant parasite and is efficiently presented by APCs upon sporozoite access to lymphoid organs. Together, our studies with Fab-treated and transgenic parasites establish a critical role for sporozoite migration to the DLN in CD8^+^ T cell priming after ID inoculation.

### Early acquisition of sporozoite-derived antigens by LN-resident DCs

**Figure 3:**
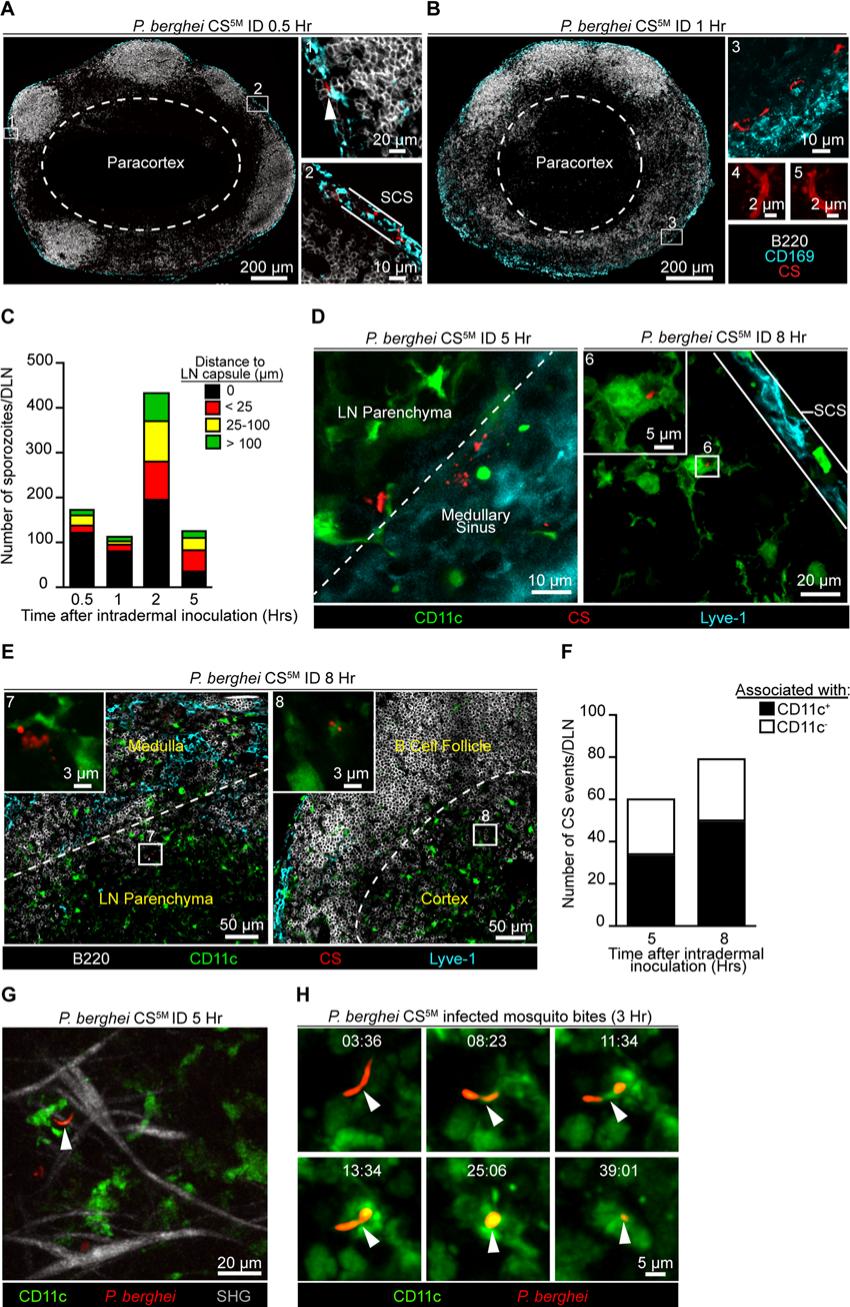
Localization, quantification, and dynamics of sporozoites in the DLN. Immunofluorescence (IF) images depicting sporozoites and CS protein in the DLNs at various time points after ID inoculation with 1×10^5^ *P*. *berghei* CS^5M^ sporozoites. A. IF images of a DLN 30 minutes post-inoculation. Box 1 and 2 indicate enlarged areas of a DLN montage. Arrowhead denotes a sporozoite in the subcapsular sinus. Subcapsular sinus abbreviated as SCS. Colors of the word labels correspond to the colors of the stains here and throughout. White dotted line demarcates the paracortex. B. IF images of a DLN 1 hour post-inoculation. Box 3 indicates a maximum intensity projection of a 30 μm z stack. Box 4 and 5 are higher magnification images of sporozoites in the DLN 1 hour after ID inoculation. C. Number of sporozoites in whole DLNs at various time points after ID inoculation. The mean from one experiment is shown and is representative of 8 similar experiments. D. IF image of whole parasites interacting with DCs 5 and 8 hours post-inoculation. White dotted line marks boundary between the medullary sinus and LN parenchyma. Box 6 is an enlarged image of a DC-associated sporozoite. E. IF images of CS-positive events in a DLN 8 hours post-inoculation. White dotted line indicates boundary between the medullary sinus and LN parenchyma or the B cell follicle and cortex. Box 7 and 8 are higher magnification images of CS-positive events associated with DCs. F. Number of CS-positive events associated with DCs in the DLNs at 5 and 8 hours post-inoculation. Mean from one experiment is shown and is representative of 2 similar experiments. G. Maximum intensity projection of a 2-photon image from a CD11c-EYFP DLN 5 hours after ID inoculation with 1×10^5^ *P*. *berghei* GFP sporozoites (pseudo-colored red). Second-harmonic generation (SHG, capsule); z stack of 30 μm. H. Time course of sporozoite uptake by a DC taken 3 hours after injection by infectious mosquito bites. Time, min:sec. See also S1 and S2 Movies.

The requirement for sporozoite-mediated delivery of antigen to the DLN was unexpected and led us to further characterize the location and behavior of parasites in this organ. *P*. *berghei* CS^5M^ sporozoites were injected ID into footpads and the popliteal LNs were harvested at various times after sporozoite inoculation, fixed, sectioned, and stained for confocal analysis. We detected sporozoites in the DLN as early as 30 minutes after sporozoite inoculation with the majority of sporozoites in close association with CD169^+^ macrophages populating the subcapsular sinus (SCS) (Fig. 3A). By 1 hour after ID inoculation, sporozoites were still present in, or immediately adjacent to, the subcapsular or medullary sinuses and displayed the characteristic crescent shape of intact sporozoites (Fig. 3B). Higher magnification revealed the presence of particulate CS staining around sporozoites in the DLN (Fig. 3B), reminiscent of the CS vesicles first detected by transmission electron microscopy [37]. The presence of intact sporozoites peaked at around 2 hours post-inoculation but declined over the next few hours (Fig. 3C). The remaining intact sporozoites were enriched in deeper interfollicular LN areas and could be found in association with DCs (Fig. 3C and D). In contrast to intact parasites, particulate CS antigen was frequently observed in the LN parenchyma and underneath the B cell follicles (Fig. 3E), with the proportion of CS-positive events associated with DCs increasing with time (Fig. 3F).

To observe the behavior and fate of sporozoites in the DLN *in vivo*, we injected GFP-expressing *P*. *berghei* CS^5M^ sporozoites into the footpads of CD11c-EYFP reporter mice [38]. Using multiphoton intravital microscopy (MP-IVM) we were able to observe motile *P*. *berghei* CS^5M^ GFP sporozoites in the superficial 100 μm of the DLN 5 hours after ID injection (Fig. 3G and S1 Movie). The sporozoites we observed in the DLN did not display gliding motility and moved more slowly than what has been reported in the skin [39]. The reduced motility we observed in the DLN is likely a constraint of the tissue microenvironment because sporozoites in the salivary glands of mosquitoes also exhibit decreased speeds as compared to sporozoites in the skin [39]. In agreement with our static imaging results showing DC-associated sporozoites, dynamic imaging revealed internalization of a live sporozoite by a DC after inoculation by infectious mosquito bites (Fig. 3H and S2 Movie). Together, these findings provide direct evidence for the migration of viable sporozoites to the DLN, their access to the parenchymal region after ID deposition, and direct uptake by LN-resident DCs.

### CD8α^+^ DCs present sporozoite antigens to CD8^+^ T cells in the DLN

**Figure 4:**
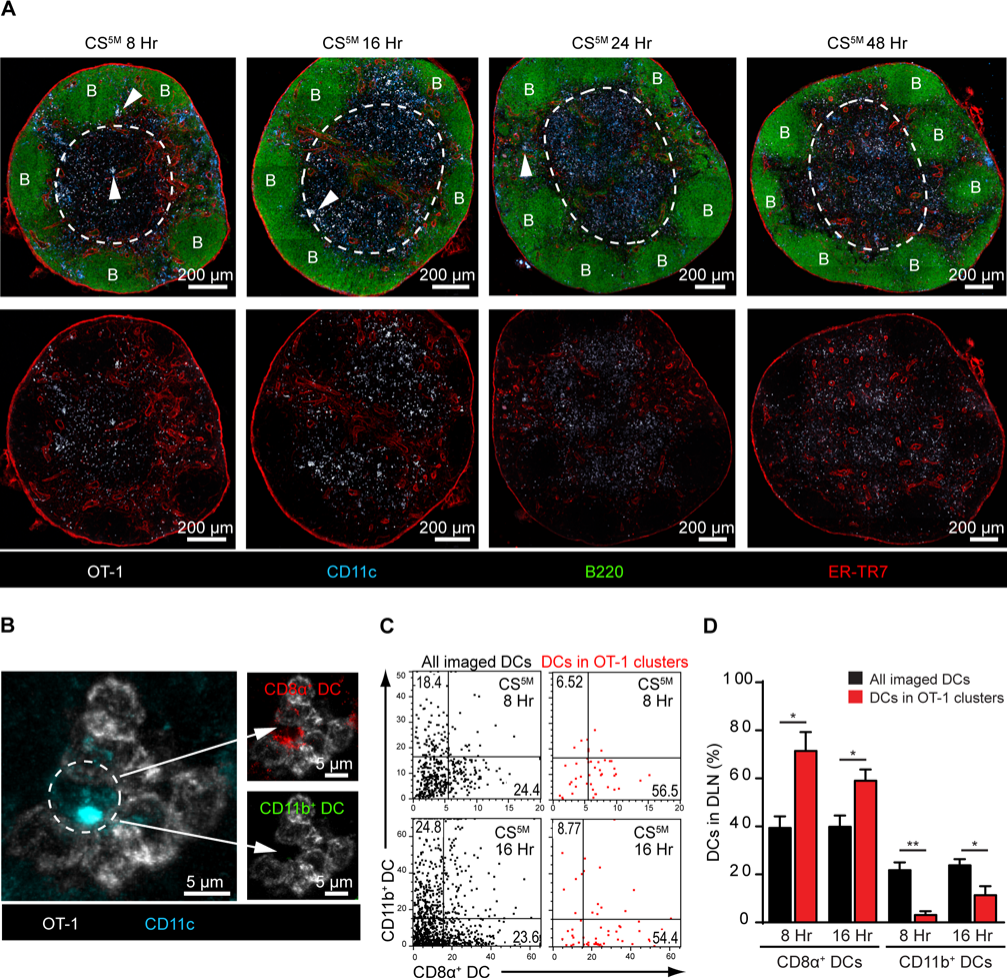
CD8α^+^ DCs present sporozoite antigens to CD8^+^ T cells in the LN cortex. A. 2×10^6^ naïve OT-1 cells were transferred to mice 1 day before ID inoculation with 1×10^5^ irradiated *P*. *berghei* CS^5M^ sporozoites. Popliteal LNs were harvested at the indicated time points and confocal images of popliteal LNs were prepared from 30 μm thick sections. White dotted line demarcates the cortex. B stands for B cell follicle. White arrowheads indicate examples of CD8^+^ T cell clusters. Representative images from 4 independent experiments with 2 mice per time point. B. Representative OT-1 cluster 8 hours after ID inoculation of *P*. *berghei* CS^5M^ sporozoites. The phenotype of the DC within the cluster (depicted with a white dotted circle) was determined by histo-cytometry; CD8α^+^ DC signal (top panel) and CD11b^+^ DC signal (bottom panel) within the cluster. C. Representative histo-cytometry scatter plots depicting the percentage of all imaged DCs (black dots) and DCs associated with OT-1 clusters (red dots) in DLNs 8 and 16 hours after ID inoculation of sporozoites. D. The percentages of OT-1 cluster-associated CD8α^+^ or CD11b^+^ DCs (red bars) vs. all imaged DCs (black bars) were quantified from 3 independent experiments with 4 DLNs/time point (mean ± SEM).

Our observations demonstrating the early acquisition of sporozoite-derived antigens by LN-resident DCs prompted us to evaluate the kinetics of CD8^+^ T cell priming in the DLN. To study antigen presentation in the DLN, we examined CD8^+^ T cell cluster formation around CD11c^+^ antigen-presenting cells (APCs) *in situ*, an established surrogate for antigen presentation [24, 25, 40–42]. In these studies, a large number of precursor cells (2.5–10×10^6^ cells) was required to visualize antigen presentation by microscopy [24, 25, 40–42]. Accordingly, we fluorescently labeled and transferred 2×10^6^ OT-1 cells to recipient mice before ID inoculation of sporozoites into the footpads. DLNs were fixed, sectioned, and stained for confocal analysis at 8, 16, 24, and 48 hours after ID inoculation (Fig. 4A). We detected OT-1 clusters in the paracortex and the boundary between the paracortex and follicles known as the cortical ridge [43] as early as 8 hours after parasite injection and these clusters increased in size and number by 16 hours. By 24 and 48 hours, the fluorescence intensity of the labeled OT-1 cells was substantially reduced, an effect likely due to dilution of the cytoplasmic label following OT-1 proliferation. Importantly, we did not observe cluster formation, nor reduced fluorescence of the labeled and transferred OT-1 cells, in mice injected with control parasites lacking the SIINFEKL epitope in CS (S3 Fig.).

To examine directly the DC subset(s) involved in the presentation of sporozoite antigens, we employed histo-cytometry, an analytical microscopy method that provides quantitatively similar results to flow cytometry but additionally gathers spatial information, allowing for the quantification of cellular interactions *in situ* [14]. Because our results indicated a critical role for LN-resident DCs in CD8^+^ T cell responses against malaria sporozoites, we designed a 6-color panel to discriminate between CD8α^+^ and CD11b^+^ LN-resident DCs on stained LN sections (S4A and B Fig.). As before, we relied on OT-1 cluster formation as a surrogate for antigen presentation and quantified DCs in direct association with OT-1 clusters (S4B Fig.). Histo-cytometric analysis revealed the presence of CD8α^+^ DCs in direct physical contact with OT-1 clusters at 8 and 16 hours after ID inoculation of sporozoites (Fig. 4B-D). At these early time points, the great majority of OT-1 clusters were associated with CD8α^+^ DCs but not CD11b^+^ DCs, indicating CD8^+^ T cell activation by the LN-resident CD8α^+^ DC subset (Fig. 4C and D). By 24 hours, OT-1 clusters were smaller but still enriched for the presence of CD8α^+^ DCs (S4C-E Fig.), whereas OT-1 clusters were nearly absent at 48 hours (S4F Fig. and Fig. 4A). These results demonstrate activation of naïve antigen-specific CD8^+^ T cells by CD8α^+^ DCs in the DLN and are fully consistent with the accepted model of temporally distinct phases of T cell priming [41].

### The early-sustained interactions between sporozoite antigen-bearing DCs and CD8^+^ T cells correlate with CD8^+^ T cell activation

**Figure 5:**
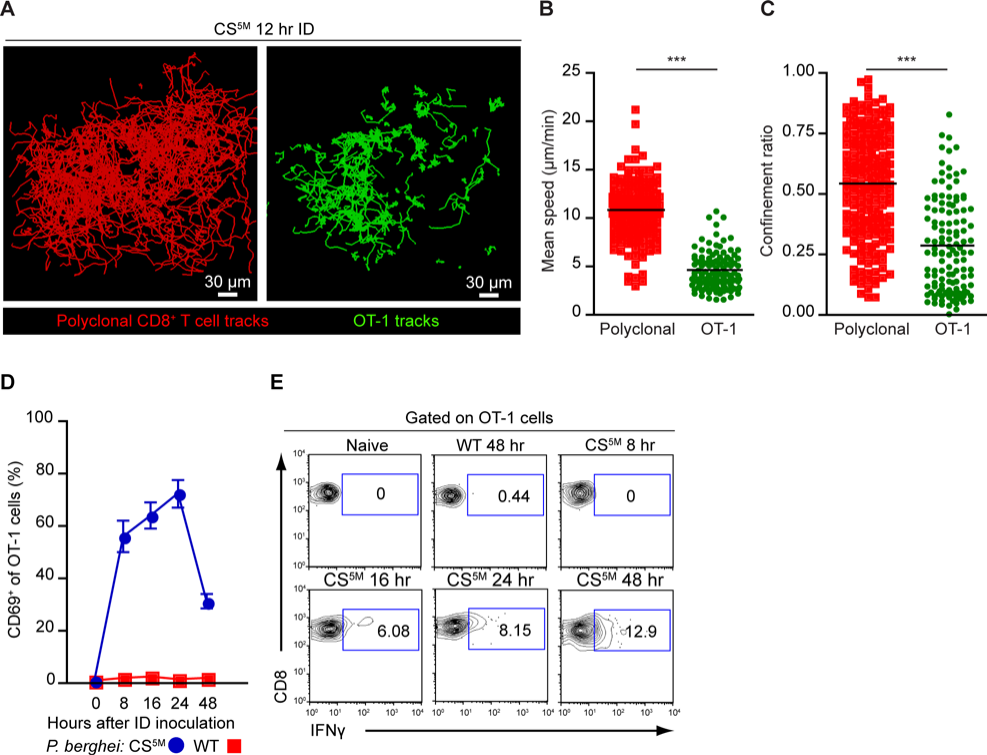
CD8^+^ T cell activation correlates with durable interactions between CD8^+^ T cells and DCs. A-C. TdTomato-expressing polyclonal CD8^+^ T cells and GFP-expressing OT-1 cells were transferred to CD11c-YFP mice 1 day before ID inoculation with 1×10^5^ irradiated *P*. *berghei* CS^5M^ sporozoites. A. Tracks of polyclonal and OT-1 cells in the popliteal LN acquired by MP-IVM 12 hours after ID inoculation of sporozoites. Mean speed analysis (B) and confinement ratio (C) of TdTomato polyclonal CD8^+^ T cells and GFP OT-1 cells *in situ*, mean ± SEM. Data points represent individual cells from one experiment, representative of four similar experiments with mean value indicated. D and E. 1×10^6^ OT-1 cells were transferred to naïve mice before ID inoculation with 1×10^5^ irradiated *P*. *berghei* CS^5M^ or *P*. *berghei* ANKA (WT) sporozoites. Single cell suspensions were prepared from popliteal LNs at the indicated time points. D. Proportion of OT-1 cells expressing CD69 based on a gate drawn on naïve OT-1 cells as controls. E. Percentage of OT-1 cells expressing IFN-γ at the indicated time points after ID inoculation. Data are representative of 2 similar experiments; mean ± SEM, n = 6/group. See also S3 Movie.

It is well established that effector formation requires long-lasting, stable interactions between T cells and DCs bearing cognate antigen [41, 44]. We therefore utilized dynamic MP-IVM to examine the duration of CD8^+^ T clusters in the DLN. OT-1 cells and polyclonal CD8^+^ T cells expressing distinct fluorescent proteins were purified and transferred to recipient mice 1–4 days prior to sporozoite injection and imaging. The superficial 200 μm of the popliteal LN accessible to 2P imaging was imaged 7–12 hours after ID inoculation of *P*. *berghei* CS^5M^ sporozoites (S3 Movie). The dynamic behavior of OT-1 cells differed from polyclonal CD8^+^ T cells in the same DLN 12 hours after ID inoculation (Fig. 5A), with OT-1 cells exhibiting a significantly reduced mean speed (Fig. 5B) and confinement ratio (Fig. 5C) as compared to polyclonal CD8^+^ T cells.

To ascertain whether these physical interactions were correlated with OT-1 activation, we transferred OT-1 cells to recipient mice and injected mice ID with *P*. *berghei* CS^5M^ sporozoites or *P*. *berghei* ANKA (WT) sporozoites that do not carry the SIINFEKL epitope in CS. Following sporozoite inoculation, DLNs were harvested and the up-regulation of CD69, an early activation marker, and the production of IFN-γ were examined on OT-1 cells by flow cytometry. By 8 hours post-inoculation with *P*. *berghei* CS^5M^ sporozoites, the majority of OT-1 cells were CD69^+^. The proportion of CD69^+^ OT-1 cells steadily increased by 16 hours and decreased 24 hours after sporozoite inoculation (Fig. 5D). Antigen-stimulated OT-1 cells produced detectable levels of IFN-γ 16 hours after *P*. *berghei* CS^5M^ injection with the proportion of IFN-γ-producing cells increasing modestly with time (Fig. 5E). In contrast, we did not observe up-regulation of CD69 or IFN-γ production by OT-1 cells in mice injected with parasites lacking the SIINFEKL epitope, *P*. *berghei* ANKA (WT) (Fig. 5D and E). These studies indicate that the early-sustained interactions between sporozoite antigen-bearing DCs and CD8^+^ T cells occurs under the conditions in which we observe robust T cell priming and activation.

### LN-resident CD8α^+^ DCs are necessary for CD8^+^ T cell priming

**Figure 6:**
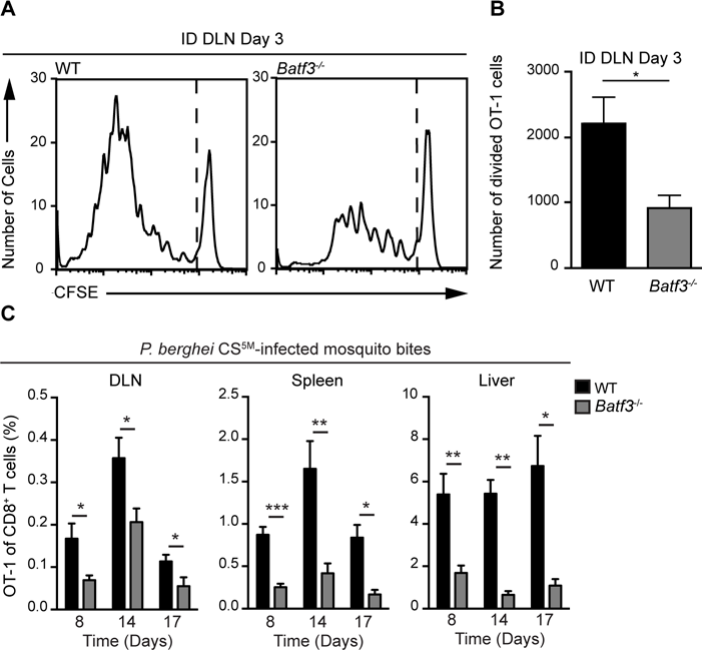
CD8α^+^ DCs are necessary for the presentation of sporozoite antigens to CD8^+^ T cells. A and B. WT and *Batf3* ^*−/−*^ mice received 1×10^6^ CFSE-labeled OT-1 cells 1 day prior to ID injection of 2×10^4^ irradiated *P*. *berghei* CS^5M^ sporozoites. DLNs were collected 3 days post-inoculation. A. Representative CFSE profiles of OT-1 cells from 1 of 3 similar experiments. B. Expansion of OT-1 cells in the DLN; mean ± SEM, n = 7/group. Data are representative of 3 similar experiments. C. 0.5–1×10^4^ naive OT-1 cells were transferred to WT and *Batf3* ^*−/−*^ mice 1 day before immunization via 20 irradiated *P*. *berghei* CS^5M^-infected mosquito bites. Responses were examined 8, 14, and 17 days post-inoculation. Proportion of CD8^+^ T cells of OT-1 origin after *P*. *berghei* CS^5M-^infected mosquito bites. Data are pooled from 2 similar experiments mean ± SEM; n = 9/group (day 8) and n = 6/group (day 14 and 17).

Given the enrichment of CD8α^+^ DCs in OT-1 clusters at time points corresponding to CD8^+^ T cell activation, we next examined the contribution of these DCs to sporozoite-specific T cell activation in *Batf3* ^−/−^ mice that possess substantial defects in the numbers of CD8α^+^ DCs [45], [46]. CFSE-labeled OT-1 cells were transferred to WT and *Batf3* ^*−/−*^ C57BL/6 mice 1 day before ID inoculation with *P*. *berghei* CS^5M^ sporozoites. The number of divided OT-1 cells was diminished by 50% in *Batf3* ^*−/−*^ C57BL/6 mice at day 3 in the DLN (Fig. 6A and B). To further investigate the requirement for CD8α^+^ DCs in the presentation of sporozoite antigens, we examined the OT-1 response in *Batf3* ^*−/−*^ C57BL/6 mice at several time points after exposure to *P*. *berghei* CS^5M^-infected mosquito bites. A significant reduction in OT-1 expansion was observed in the DLN, spleen, and liver of *Batf3* ^*−/−*^ C57BL/6 mice at day 8, 14, and 17 (Fig. 6C and S5A Fig.). Following a report demonstrating the potential presence of CD8α^+^ DCs in the DLNs of *Batf3* ^*−/−*^ C57BL/6 mice at steady state and in additional organs after administration of IL-12 [47], we repeated the priming experiments in *Batf3* ^*−/−*^ mice on the 129/SvEV background in which CD8α^+^ DCs and langerin^+^ dermal DCs are largely absent and observed a 75% reduction in OT-1 expansion (S5B and C Fig.). Besides the important role for CD8α^+^ DCs in the activation of CS-specific CD8^+^ T cells, we also found a reduction in CD8^+^ T cell priming in the absence of CD169^+^ macrophages (S6 Fig.). Despite a reduction in OT-1 expansion in DT-treated CD169-DTR mice, we did not observe OT-1 cluster formation around CD169^+^ macrophages lining the subcapsular or medullary sinuses (Fig. 4) and therefore conclude that CD169^+^ sinus-associated macrophages may play an indirect role in the generation of optimal CD8^+^ T cell responses.

## Discussion

**Figure 7:**
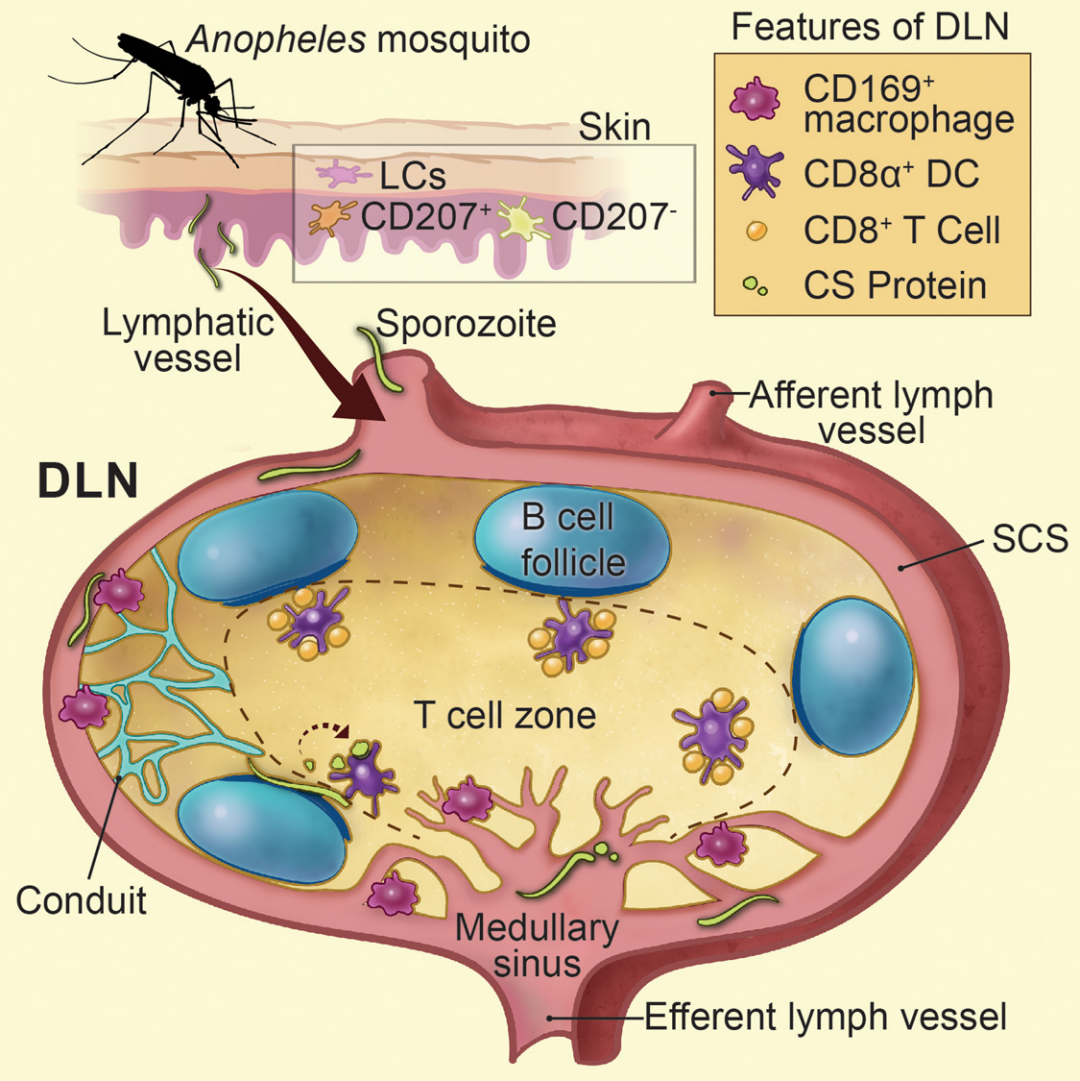
Summarizes Figs. 1–6 as a model. Sporozoite-mediated delivery of malaria antigens to the DLNs is required for CD8^+^ T cell priming. Anopheline mosquitoes inject sporozoites into the skin of their mammalian host, a tissue populated by epidermal Langerhans cells, langerin^+^ dermal DCs (CD207^+^), and langerin^-^ dermal DCs (CD207^-^). Following sporozoite inoculation into the dermis, a proportion of sporozoites enter the DLNs via the afferent lymphatic vessel and are found in close association with CD169^+^ macrophages lining the subcapsular and medullary sinuses. LN-resident DCs directly sample and phagocytose sporozoites and sporozoite-derived CS particles within the LN parenchyma. Sporozoite-specific CD8^+^ T cells are primed by LN-resident CD8α^+^ DCs residing along the cortical ridge and the T cell zone of the DLN as early as 8 hours after immunization. Printed with permission from Heidi Sinsel, 2014.

*Plasmodium* sporozoites are introduced into the dermis of their mammalian host by infectious mosquito bites (reviewed in [26]), and a proportion of these sporozoites enter the lymphatics early after inoculation [4, 39, 48]. Following this natural route of parasite delivery, CS-specific CD8^+^ T cells are primed in the LNs draining the site of sporozoite inoculation [4]. Here, we present clear evidence indicating that direct parasite access to the DLN is required for the induction of CD8^+^ T cells directed against *P*. *berghei* CS (Fig. 7). Two independent experimental approaches were used to establish the immunological significance of parasites in the DLN. First, immobilization of sporozoites through pre-treatment with a CS-specific mAb, or monovalent Fab fragments derived from this antibody, resulted in reduced parasite burdens in the DLN and severely diminished CD8^+^ T cell responses directed against an antigenic determinant in CS. Second, mutant parasites with a defect in their ability to exit the skin and enter the DLNs also generated poor CD8^+^ T cell responses when injected ID. The relationship between live, motile sporozoites and the induction of robust CD8^+^ T cell responses is further supported by the observation that when dead sporozoites are injected ID they do not reach the DLN [39] and do not induce CD8^+^ T cell responses [4]. Reduced CS-specific CD8^+^ T cell responses were also observed in the spleen following IV injection of dead sporozoites [49]. This was surprising because IV injected sporozoites have direct access to the spleen, the site of CTL activation after IV immunization [50]. Therefore, the importance of live sporozoites in the development of CD8^+^ T cells likely extends beyond efficient drainage to lymphoid organs and may indicate a requirement for sporozoite motility within these organs.

Sporozoites are renowned for their ability to migrate through many different cell types [51–54]. As sporozoites glide they release trails of CS comprised of 25–90 nm beadlike particles [37]. Our confocal analysis revealed the accumulation of particulate CS around intact parasites 1 to 2 hours after sporozoite injection. Based on the nature of CS staining and the timing of our *in vivo* observations, we hypothesize that particulate CS is actively shed by motile sporozoites in the DLN and may represent a source of antigen for cross-presentation by LN-resident DCs. In addition, we frequently observed sporozoites underneath the B cell follicles and immediately adjacent to the cortical ridge of the DLN. Therefore, live sporozoites may provide a source of CS that can be directly sampled and presented by DCs in the cortical ridge and paracortex of the DLN, the location of CD8^+^ T cell priming in our model. In further support of this idea, we visualized abundant stores of DC-associated CS in the DLN, which could derive from internalization of shed CS or direct uptake of live sporozoites as shown in S2 Movie.

The observation of CD8^+^ T cell cluster formation and IFN-γ production as early as 8 and 16 hours post-immunization, respectively, is in agreement with a limited role for migratory skin-DCs since these cells require anywhere from 16 hours to 5 days to migrate to the DLN [15, 55]. In addition, we observed robust CD8^+^ T cell priming in the absence of langerin^+^ dermal DCs and Langerhans cells using the MuLangerin-DTR/EGFP mouse model. However, it should be noted that migratory langerin^-^ dermal DCs remain in the skin of DT-treated MuLangerin-DTR/EGFP mice [15] and two vaccine models have demonstrated the involvement of these cells in the presentation of skin-derived antigens to CD8^+^ T cells [56, 57]. Nonetheless, the location of CD8^+^ T cell cluster formation complements the diminished priming we observed in *Batf3-*deficient mice because CD8α^+^ DCs are known to populate the cortical ridge and paracortex of the DLN [14, 43]. Importantly, histo-cytometric analysis of LN sections revealed a significant enrichment of CD8α^+^ DCs in association with OT-1 clusters at time points corresponding to T cell activation. Although sporozoites can invade and develop within LN-resident CD11b^+^ myeloid cells [58], our studies demonstrate a pivotal role for CD8α^+^ DCs in the presentation of sporozoite antigens to CD8^+^ T cells.

The reduced CD8^+^ priming we observed in mice lacking CD169^+^ macrophages further emphasizes the importance of LN-resident APCs in liver stage immunity. Because OT-1 clusters were not directly associated with CD169^+^ macrophages, we conclude that these cells play an indirect role in CD8^+^ T cell priming. CD169^+^ macrophages may facilitate CD8^+^ T cell priming via the capture and transfer of antigen to cross-presenting DCs, a phenomenon shown to take place in the spleen following delivery of blood-borne antigens [59], or in a cytokine-dependent manner as demonstrated for LN-resident innate lymphoid cells [60]. Regardless of the mechanism, elucidating the contribution of CD169^+^ macrophages to liver stage immunity remains a key direction for future research.

Others have shown a requirement for CD8α^+^ DCs in the presentation of *Plasmodium* blood and liver stage antigens [8, 9, 61–62]. Our study now documents the presentation of sporozoite antigens by LN-resident CD8α^+^ DCs *in situ* while providing a potential explanation—direct parasite access to these DC subsets—for the superior immunogenicity of live versus dead parasites. We have based our analysis on the immunodominant CS protein [63]; however, it is possible that CD8α^+^ DCs present additional liver stage antigens in the liver draining lymph nodes or the liver site of infection, as shown previously [9]. Because liver stage antigens elicit broad, cross-stage protection [64–65], an important future direction will be to evaluate the contribution of CD8α^+^ DCs to the generation of CD8^+^ T cells against these antigens. The recent success of the IV administered live, attenuated *P*. *falciparum* sporozoite vaccine (PfSPZ) [16], along with the discovery of a human homologue of murine CD8α^+^ DCs [66–68], suggests that antigen delivery to this DC subset may also be critical for CTL responses in humans. Because large-scale vaccination with the IV administered PfSPZ vaccine is likely infeasible, there is a growing need to improve the efficacy of non-IV routes, which have been shown to be inefficient in mice [6, 69], and suboptimal in humans [70]. One promising strategy is to use adjuvants to reduce the number of irradiated sporozoites required to confer sterile immunity after ID immunization [69]. Given the vital role for CD8α^+^ DCs in the presentation of malaria antigens, and the unique expression of pattern recognition receptors among different DC subsets [71–72], it will be interesting to determine whether adjuvant activation of CD8α^+^ DCs can provide better protection after ID immunization.

The antagonistic effect of anti-sporozoite antibodies on CD8^+^ T cell priming reported here and elsewhere [7] suggests that humoral immunity may be generated at the expense of cellular immunity. Therefore, it may be difficult to generate robust CD8^+^ T cell responses in individuals with high-titer anti-CS antibodies. This assertion is based on the following observations: (1) antibody-treated parasites are immobilized in the skin [35]; (2) immobilized parasites are cleared by CD11b^+^ leukocytes in the skin [51]; and (3) skin-resident CD11b^+^ leukocytes are largely dispensable for CS-specific CD8^+^ T cell responses (reported here). Thus, a major challenge for the malaria vaccine effort will be to incorporate the very behaviors required for successful parasite infection—cell traversal and cell invasion—into a vaccine that elicits long-lasting, protective immunity.

A critical question remains: Why do malaria parasites go to the DLN? This question gains additional significance when one considers that the LN is dispensable for parasite development but imperative for host immune responses. As there is no evidence for sporozoite chemotaxis to lymphatic vessels, parasite entry into the lymphatic vessels in the skin appears to be an accidental but migration-dependent process. Importantly, this phenomenon is not unique to rodent malaria models but was also observed in early human studies [73]. Together, these results underscore the importance of live, motile sporozoites in the induction of CD8^+^ T cell responses and thus, have implications for whole parasite vaccine efforts.

## Materials and Methods

### Mice

All animal procedures were approved by the Institutional Animal Care and Use Committee of Johns Hopkins University (Protocol number: MO13H123) following the NIH guidelines for animal housing and care or performed according to protocols approved by the NIAID and NIH Animal Care and Use Committee. A detailed list of mouse strains can be found in the S1 Methods section.

### Parasites and immunizations

*P*. *berghei* CS^5M^ parasites [7] and *P*. *berghei* ANKA parasites expressing GFP under the HSP70 promoter (*P*. *berghei*-ConF) have been described previously [51]. *P*. *berghei* CS^5MΔN^ parasites were created by transfection of *P*. *berghei* ANKA with a linearized pR-CSRepΔN5M plasmid. The pR-CSRepΔN5M plasmid was generated by ligating an EagI-PacI digestion product of the pCSRep5M plasmid into the pR-CSRepΔN plasmid containing the N-terminal deletion of the *CSP* locus and a drug selection cassette [36]. Mutant parasites were selected by pyrimethamine and cloned by limiting dilution. Sporozoites were dissected by hand from *Anopheles stephensi* salivary glands, radiation-attenuated in a cesium radiator at 20,000 rad., and injected ID with a nanofil syringe equipped with a 33 gauge needle (World Precision Instruments, Sarasota, FL). Alternatively, mice were injected by the bites of 20–30 irradiated day 21 *P*. *berghei* CS^5M^-infected mosquitoes. *P*. *berghei* CS^5M^-GFP parasites were generated by crossing *P*. *berghei* CS^5M^ parasites with *P*. *berghei* ConF parasites, followed by selection as described previously [74].

### Quantification of parasite RNA in DLN

At the indicated time points, LNs were harvested in TRIzol Reagent (Life Technologies) and parasite RNA was extracted according to the manufacturer’s instructions. Parasite burden was measured by RT-PCR using primers that recognize *P*. *berghei* specific sequences within the 18S rRNA and SYBR Green (Applied Biosystems) as outlined previously [75]. Parasite burdens were normalized with GAPDH expression.

### Lymphocyte isolation

Single cell suspensions were prepared by grinding the spleen and DLNs between the rough sides of 2 microscope slides and by filtering with nylon mesh. Liver homogenates were passed over a 35% percoll gradient and filtered through nylon mesh. Lymphocytes were resuspended in DMEM supplemented with 10% heat-inactivated fetal bovine serum (FBS), 50 mM sodium bicarbonate, 2mM glutamine, 100 U/ml penicillin, 100 μg/ml streptomycin, 25 mM HEPES.

### Adoptive CD8^+^ T cell transfer

OT-1 T cells or polyclonal control CD8^+^ T cells were sorted with a MACS CD8-negative selection kit (Miltenyi). Prior to cell transfer, the quality of purification was verified by flow cytometry using antibodies against Vα2 and CD8. Cells of approximately 90% purity were stained and transferred 1 day before sporozoite inoculation.

### Quantification of CD8^+^ T cell expansion *in vivo*

5×10^3^ OT-1 (CD45.1^+^) cells were transferred IV to recipient (CD45.2^+^) mice 1 day before needle or mosquito bite inoculation of irradiated sporozoites. In some experiments, expansion of OT-1 cells was measured by flow cytometry on days 8, 10, 14, or 17. In other experiments, 1–2×10^6^ OT-1 cells were labeled with 10 μM CFSE (Invitrogen) or 100 μM CellTracker Blue CMF_2_HC (Invitrogen) and transferred IV to recipient mice. OT-1 cluster formation and proliferation were evaluated 8–72 hours after sporozoite inoculation.

### Confocal microscopy and histo-cytometry

Detailed methods for sample preparation, imaging, and data analysis are available in the S1 Methods section.

### Multi-photon intravital imaging

Mice were anesthetized and popliteal LNs were exposed. MP-IVM was performed by a protocol modified from a previous report [76].

### Analysis

Flow cytometric data was analyzed with FlowJo software (TreeStar). Raw imaging data were processed and analyzed with Imaris software (Bitplane). Differences between two groups were compared using a Student’s t test (two-tailed) for normal distributions or Mann-Whitney test for non-normal distributions. One-way analysis of variance with Tukey post-test was used to compare differences between more than two groups. (ns = not significant, * = P < 0.05, ** = P < 0.01, *** = P < 0.001)

## Supporting Information

S1 Fig Depletion of langerin^+^ cells in the skin and DLNs of MuLangerin-DTR/EGFP C57BL/6 mice. Heterozygous MuLangerin-DTR/EGFP mice on the C57BL/6 background received a single IP injection of 1 ug DT. Whole ear mounts and DLNs were collected at the indicated time points after DT injection. A. Whole ear mounts were fixed and stained with an anti-langerin antibody and appropriate secondary antibody. Ears of 4 mice per time point were examined for the presence of langerin^+^ cells and z stacks were taken of representative sections. Immunofluorescence images depict a maximum intensity projection of 27 um. No langerin^+^ events were present in the ears of mice analyzed 2 days after DT. Langerin^+^ DCs were not present in the majority of ears scanned 6 days after DT treatment; however, a rare langerin^+^ DC was encountered (inset) and is presumably a langerin^+^ CD103^+^ DC. Dim langerin^+^ events were present in all ears examined at 14 days after DT, depicted in the rightmost panel. B. DLNs were collected from individual mice at different time points after DT treatment. Percentages of cells expressing langerin among CD11c^+^ 1-Ab^+^ cells from pooled DLNs. C. Kinetics of the repopulation of skin-emigrant langerin^+^ DCs in the DLNs (mean ± SEM, n = 2–3 mice/time point). Representative data from two independent experiments are shown. D. MuLangerin-DTR/EGFP and C57BL/6 mice received a single IP injection of DT (1 μg). 24 hours after DT treatment, 5×10^3^ naive OT-1 cells were adoptively transferred to recipient mice. Mice were immunized through the bites of 20 irradiated *P*. *berghei* CS^5M^-infected mosquitoes 1 day after cell transfer and 2 days after DT treatment. Number of OT-1 cells recovered 10 days after sporozoite inoculation. Data are pooled from 2 similar experiments (mean ± SEM; n = 9–10/group).
(TIF)

S2 Fig Generation and *in vivo* phenotype of *P*. *berghei* CS^5MΔN^ parasites. A. Scheme of the strategy used for gene targeting of the replacement *CSP* locus. An EagI/PacI fragment containing the model H-2K^b^-restricted epitope SIINFEKL was ligated into a transfection plasmid containing a mutant *CSP* locus with a deletion in the N-terminus. The native *CSP* was replaced with the mutated *CSP* via double homologous recombination. B. To verify deletion of the N-terminus of *CSP*, PCR was performed with a forward primer upstream of the N-terminal deletion (F129) and a reverse primer within the *CSP* locus (R1484), yielding a 1.1 Kb product. pRCS-CS^5M^ is DNA from a plasmid with an intact *CSP* locus, pRCSRepΔN is DNA from a plasmid with a truncated *CSP* locus, CS^5M^ is genomic DNA from parasites with an intact *CSP* locus, and CS^5MΔN^ is genomic DNA from parasites with a N-terminal deletion in *CSP*. C. To verify insertion of the SIINFEKL epitope and 3’ integration, PCR was performed using a forward primer containing the SIINFEKL epitope (DNOva) and a reverse primer located in the 3’ UTR of the *CSP* locus (CS4), yielding a 1.3 Kb product. CS^5MΔN^ is genomic DNA from parasites with a truncated *CSP* locus containing the model H-2K^b^-restricted epitope SIINFEKL. WT is genomic DNA from *P*. *berghei* ANKA parasites. D. Naïve mice were injected ID with 5×10^3^ *P*. *berghei* CS^5M^ or *P*. *berghei* CS^5MΔN^ sporozoites. DLNs were collected at 0.5, 2, and 5 hours after challenge. Total RNA was isolated and parasite copies were quantified using primers that recognize parasite-specific sequences within the 18S rRNA. Parasite burdens were pooled from 3 similar experiments and normalized with GAPDH; n = 15/group, mean ± SEM. E and F. Mice received 2×10^6^ CFSE-labeled naïve OT-1 cells 1 day prior to ID injection of 2 x 10^4^ irradiated *P*. *berghei* CS^5M^ or *P*. *berghei* CS^5MΔN^ sporozoites. DLNs were collected 3 days post-inoculation. E. Representative CFSE profiles of OT-1 cells from 1 of 2 similar experiments. F. Number of divided OT-1 cells in the DLN, (mean ± SEM, n = 3/group), data representative of 2 similar experiments. G. Naïve mice were injected IV with 2×10^4^ *P*. *berghei* CS^5M^ or *P*. *berghei* CS^5MΔN^ sporozoites. Livers were collected 40 hours after sporozoite injection. Parasite burdens were quantified as described in panel D. Data are representative of 3 similar experiments (n = 3/group, mean ± SEM).
(TIF)

S3 Fig *P*. *berghei* ANKA (WT) sporozoites do not induce CD8^+^ T cell cluster formation in the DLN. A. 2×10^6^ OT-1 cells were transferred to naïve mice 24 hours before ID inoculation with 1×10^5^ irradiated *P*. *berghei* ANKA (WT) sporozoites. Popliteal LNs were harvested at the indicated time points and confocal images of DLNs were prepared from 30 μm thick sections. White dotted line demarcates the cortex. B stands for B cell follicle. Representative images from 1 experiment with 2 mice per time point. B. Higher magnification of DLN section 16 hours after ID inoculation with 1×10^5^ irradiated *P*. *berghei* ANKA (WT) sporozoites.
(TIF)

S4 Fig Histo-cytometric analysis of DC subsets presenting sporozoite antigens to CD8^+^ T cells in the DLN. A. Confocal images of a representative LN section stained with a 6-color panel consisting of antibodies directed against CD3, CD8, CD45.1 (OT-1), CD11b, CD11c, and MHC II. B. Schema representing the theory behind histo-cytometry. Because CD8α is expressed on CD8^+^ T cells as well as a subset of DCs, we first had to generate a DC-specific channel by voxel gating on the CD11c^+^ CD3^-^ CD45.1^-^ population (Step 1). Once we obtained a ‘Gated DC’ channel, we examined the mean voxel intensities for the CD8 and CD11b channels within DCs (CD3^-^ CD45.1^-^ CD11c^+^) and generated new channels corresponding to CD8α^+^ and CD11b^+^ DC subsets (Step 2). To identify which DCs were presenting sporozoite antigens to the OT-1 cells, we generated a unique channel corresponding to OT-1 clusters using the imaging software Imaris (Step 3). We exported our imaging data to FlowJo and used the ‘OT-1 cluster’ channel to gate on DC populations within OT-1 clusters (Step 4). C-F. 2×10^6^ OT-1 (CD45.1^+^) cells were transferred to naïve mice 1 day before ID inoculation with 1×10^5^ irradiated *P*. *berghei* CS^5M^ sporozoites. Popliteal LNs were harvested at the indicated time points, stained with the 6-color panel outlined in A, and imaged with a confocal microscope. C. IF image of OT-1 clusters taken 24 hours after ID inoculation of *P*. *berghei* CS^5M^ sporozoites. White circles highlight representative OT-1 clusters. Arrowhead indicates OT-1 cluster-associated CD8α^+^ DC. D. Representative histo-cytometry scatter plots depicting the percentage of all imaged DCs (black dots) and DCs associated with OT-1 clusters (red dots) in DLNs 24 hours after ID inoculation of sporozoites. E. The percentages of cluster-associated CD8α^+^ or CD11b^+^ DCs (red bars) vs. all imaged DCs (black bars) were quantified from 2 independent experiments with 3 DLNs/time point (mean ± SEM). F. Representative image of robust proliferation of OT-1 cells 48 hours after ID inoculation of sporozoites.
(TIF)

S5 Fig *Batf3* ^−/−^ mice mount reduced CD8^+^ T cell responses against malaria sporozoites. A. 0.5–1×10^4^ naive OT-1 cells were transferred to WT and *Batf3* ^*−/−*^ mice 1 day before immunization via 20 irradiated *P*. *berghei* CS^5M^-infected mosquito bites. Responses were examined 8, 14, and 17 days post-inoculation. Number of OT-1 cells in the DLNs, spleen, and liver of immunized mice. Data are pooled from 2 similar experiments mean ± SEM; n = 9/group (day 8) and n = 6/group (day 14 and 17). B and C. To reduce the likelihood of OT-1 rejection by 129 SvEV recipient mice, we crossed OT-1 TCR transgenic mice on the C57BL/6 background to 129 SvEV mice. OT-1 cells were purified from 129 SvEV/C57BL/6 mice, CFSE-labeled, and adoptively transferred to *Batf3* ^−/−^ mice on the 129 SvEV background or 129 SvEV control mice. Mice were immunized with 2×10^4^ irradiated *P*. *berghei* CS^5M^ sporozoites 24 hours after transfer of 1×10^6^ OT-1 cells. DLNs were harvested 72 hours after sporozoite immunization and analyzed for CFSE dilution. B. Representative plots from WT and *Batf3* ^−/−^ mice. C. Number of divided OT-1 cells pooled from 2 similar experiments (mean ± SEM; n = 6–7/group).
(TIF)

S6 Fig Impaired expansion of OT-1 cells following DT depletion of CD169^+^ macrophages. The skin-DLNs of WT and CD169-DTR mice were harvested 2 and 5 days after a single IP injection of 1 ug DT. Cells were gated on single, live cells. A. Percentage of migratory (CD11c^Int^ 1-Ab^Hi^) and resident (CD11c^Hi^ 1-Ab^Int^) DCs from the skin-DLNs from an individual mouse 48 hours after DT treatment. B. Number of conventional DCs from the skin-DLNs of individual WT and CD169-DTR mice; representative of 3–4 mice/group. C. Percentage of CD169^+^ cells in the skin-DLNs of WT and CD169-DTR mice 2 and 5 days after DT treatment. D. Number of CD169^+^ CD11c^Int^ cells from the skin-DLNs of individual WT and CD169-DTR mice and representative of 3–4 mice/group. E and F. Mice were injected with 1 ug DT one day prior to adoptive transfer of 1×10^6^ purified, CFSE-labeled OT-1 cells to WT and CD169-DTR mice. 24 hours after cell transfer and 48 hours after DT treatment, mice were inoculated with 2×10^4^ irradiated *P*. *berghei* CS^5M^ sporozoites ID. DLNs were harvested 72 hours after sporozoite immunization and analyzed for CFSE dilution. E. Representative CFSE dilution plots from WT and CD169-DTR mice treated with DT. F. Number of divided OT-1 cells (mean ± SEM; n = 3–5/group). The data presented here are from one independent experiment and are representative of 2–3 similar experiments.
(TIF)

S1 Movie Dynamic behavior of P. berghei sporozoites *in vivo*. 2 photon (2P) movie of a DLN from a CD11c-EYFP mouse (green) captured 5 hours after ID inoculation with 1×10^5^ *P*. *berghei* CS^5M^ GFP sporozoites (pseudo-colored red). 30 μm z stacks were acquired every 40 seconds to construct a 40 minute movie. LN capsule (gray) revealed by second-harmonic generation. Representative of 4 similar experiments.
(MP4)

S2 Movie Direct uptake of a sporozoite by a DC. 2 photon (2P) movie of a DLN from a CD11c-EYFP mouse (green) captured 3 hours after ID inoculation of *P*. *berghei* CS^5M^ GFP sporozoites (pseudo-colored red) by infectious mosquito bites. Mice were injected subcutaneously with Qdot-705 for lymph labeling (blue). 30 μm z stacks were acquired every 40 seconds to construct a 40 minute movie.
(MP4)

S3 Movie CS-specific CD8^+^ T cells form stable contacts with CD11c^+^ DCs in the DLN. TdTomato-expressing polyclonal CD8^+^ T cells (red) and GFP-expressing OT-1 cells (green) were transferred to CD11c-YFP mice (yellow) 1 day before ID inoculation with 1×10^5^ *P*. *berghei* CS^5M^ sporozoites. Shown here is a 2P movie from the DLN of a CD11c-EYFP mouse acquired 12 hours after ID inoculation with sporozoites. A 40 minute movie was constructed from 60 μm z stacks acquired every 40 seconds. Representative of 4 similar experiments.
(MP4)

S1 Methods A comprehensive list of mouse strains, antibodies, and primers can be found in the S1 Methods section of the manuscript. In addition, detailed protocols for immunofluorescence, flow cytometry, and parasite cloning are included in this section.
(DOCX)
