# Supplementary figures and images for: Lymph-Node Resident CD8α+ Dendritic Cells Capture Antigens from Migratory Malaria Sporozoites and Induce CD8+ T Cell Responses
Source: PLoS Pathog. 2015 Feb 6;11(2):e1004637. doi: 10.1371/journal.ppat.1004637 (PMC4450069; doi:10.1371/journal.ppat.1004637)

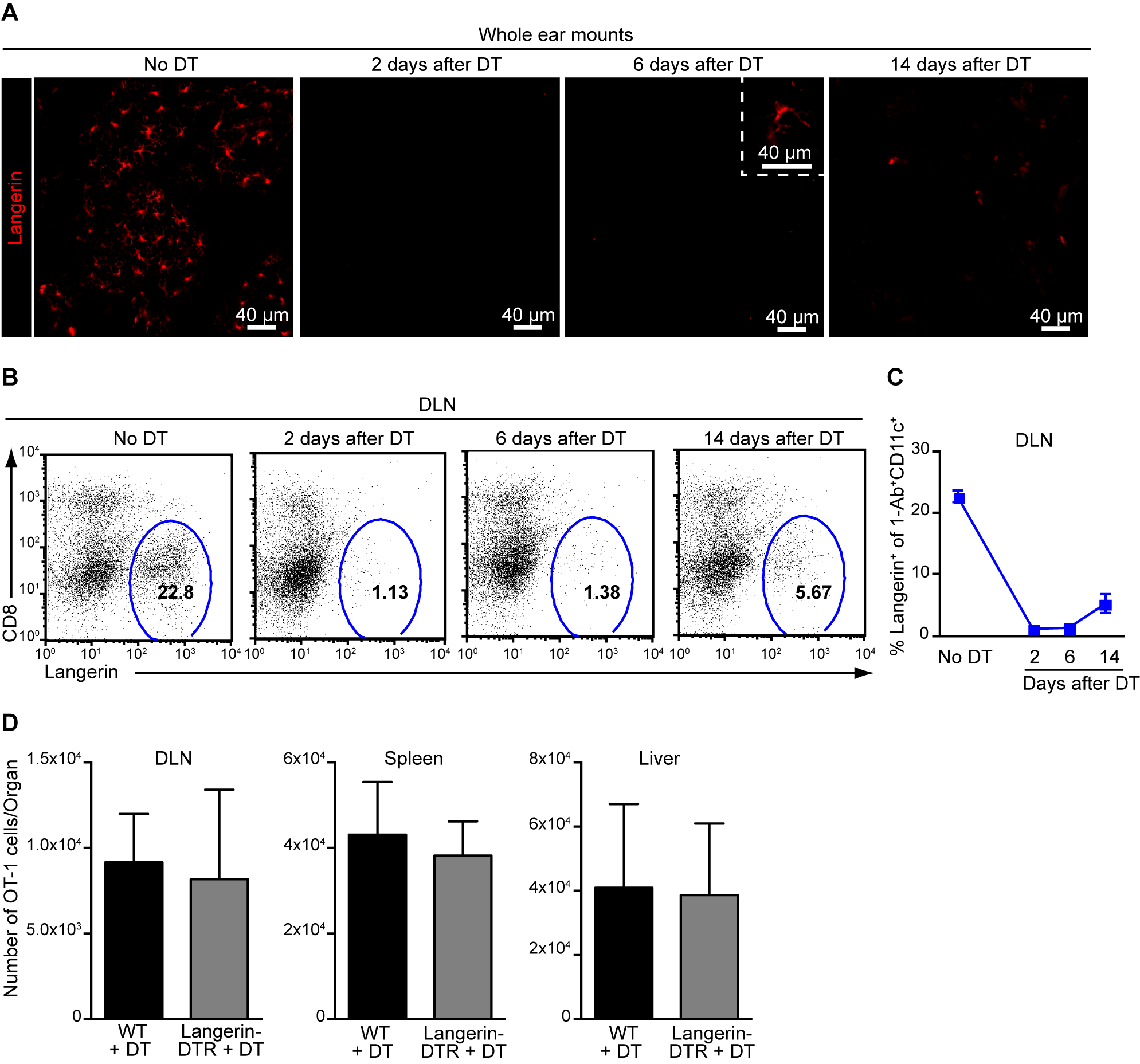

Supplement: S1 Fig — Heterozygous MuLangerin-DTR/EGFP mice on the C57BL/6 background received a single IP injection of 1 ug DT. Whole ear mounts and DLNs were collected at the indicated time points after DT injection. A. Whole ear mounts were fixed and stained with an anti-langerin antibody and appropriate secondary antibody. Ears of 4 mice per time point were examined for the presence of langerin+ cells and z stacks were taken of representative sections. Immunofluorescence images depict a maximum intensity projection of 27 um. No langerin+ events were present in the ears of mice analyzed 2 days after DT. Langerin+ DCs were not present in the majority of ears scanned 6 days after DT treatment; however, a rare langerin+ DC was encountered (inset) and is presumably a langerin+ CD103+ DC. Dim langerin+ events were present in all ears examined at 14 days after DT, depicted in the rightmost panel. B. DLNs were collected from individual mice at different time points after DT treatment. Percentages of cells expressing langerin among CD11c+ 1-Ab+ cells from pooled DLNs. C. Kinetics of the repopulation of skin-emigrant langerin+ DCs in the DLNs (mean ± SEM, n = 2–3 mice/time point). Representative data from two independent experiments are shown. D. MuLangerin-DTR/EGFP and C57BL/6 mice received a single IP injection of DT (1 μg). 24 hours after DT treatment, 5×10^3^ naive OT-1 cells were adoptively transferred to recipient mice. Mice were immunized through the bites of 20 irradiated P. berghei CS5M-infected mosquitoes 1 day after cell transfer and 2 days after DT treatment. Number of OT-1 cells recovered 10 days after sporozoite inoculation. Data are pooled from 2 similar experiments (mean ± SEM; n = 9–10/group). (TIF) [file ppat.1004637.s001.tif]

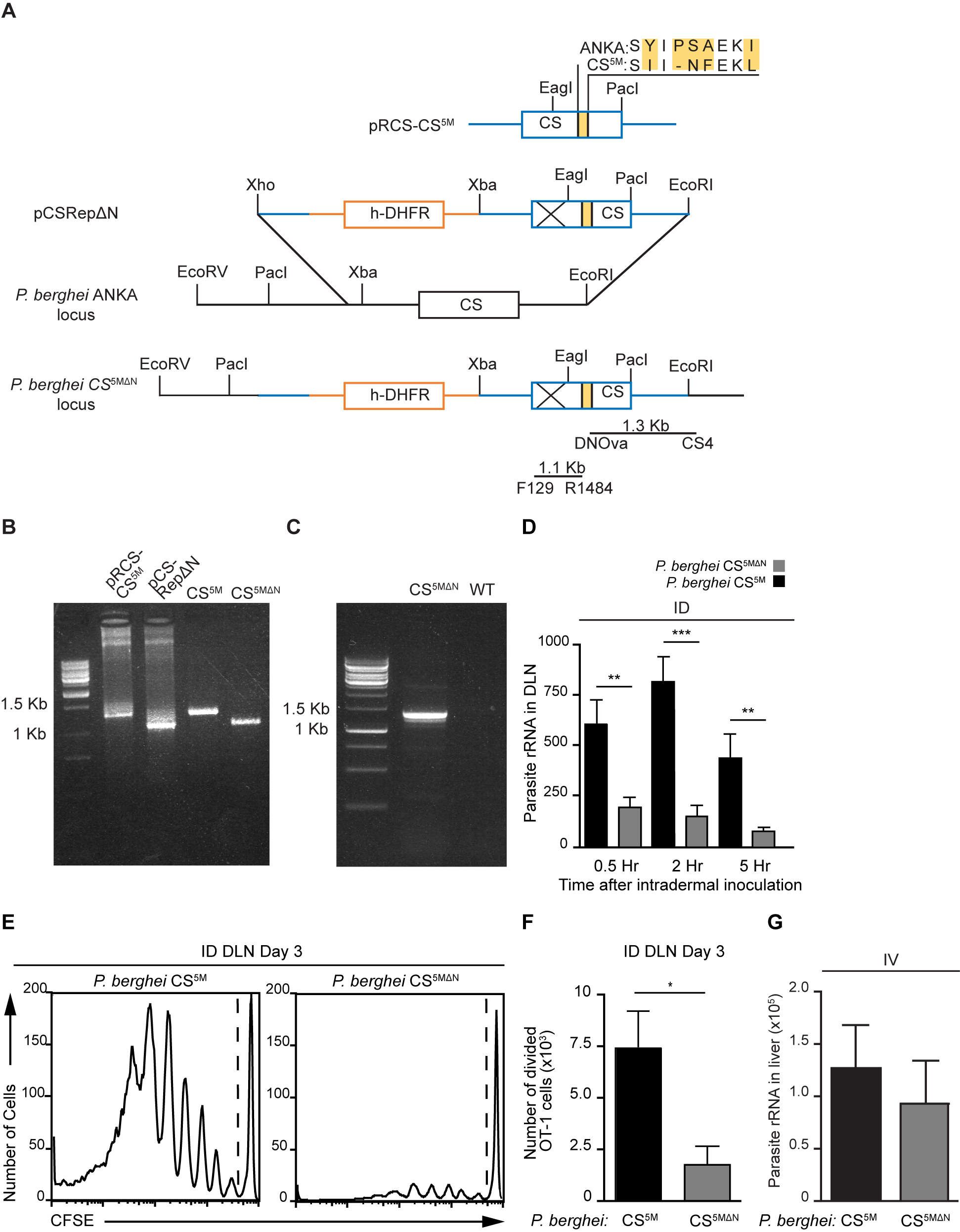

Supplement: S2 Fig — A. Scheme of the strategy used for gene targeting of the replacement CSP locus. An EagI/PacI fragment containing the model H-2Kb-restricted epitope SIINFEKL was ligated into a transfection plasmid containing a mutant CSP locus with a deletion in the N-terminus. The native CSP was replaced with the mutated CSP via double homologous recombination. B. To verify deletion of the N-terminus of CSP, PCR was performed with a forward primer upstream of the N-terminal deletion (F129) and a reverse primer within the CSP locus (R1484), yielding a 1.1 Kb product. pRCS-CS5M is DNA from a plasmid with an intact CSP locus, pRCSRepΔN is DNA from a plasmid with a truncated CSP locus, CS5M is genomic DNA from parasites with an intact CSP locus, and CS5MΔN is genomic DNA from parasites with a N-terminal deletion in CSP. C. To verify insertion of the SIINFEKL epitope and 3’ integration, PCR was performed using a forward primer containing the SIINFEKL epitope (DNOva) and a reverse primer located in the 3’ UTR of the CSP locus (CS4), yielding a 1.3 Kb product. CS5MΔN is genomic DNA from parasites with a truncated CSP locus containing the model H-2Kb-restricted epitope SIINFEKL. WT is genomic DNA from P. berghei ANKA parasites. D. Naïve mice were injected ID with 5×10^3^ P. berghei CS5M or P. berghei CS5MΔN sporozoites. DLNs were collected at 0.5, 2, and 5 hours after challenge. Total RNA was isolated and parasite copies were quantified using primers that recognize parasite-specific sequences within the 18S rRNA. Parasite burdens were pooled from 3 similar experiments and normalized with GAPDH; n = 15/group, mean ± SEM. E and F. Mice received 2×10^6^ CFSE-labeled naïve OT-1 cells 1 day prior to ID injection of 2 x 10^4^ irradiated P. berghei CS5M or P. berghei CS5MΔN sporozoites. DLNs were collected 3 days post-inoculation. E. Representative CFSE profiles of OT-1 cells from 1 of 2 similar experiments. F. Number of divided OT-1 cells in the DLN, (mean ± SEM, n = 3/group), data representative of 2 [file ppat.1004637.s002.tif]

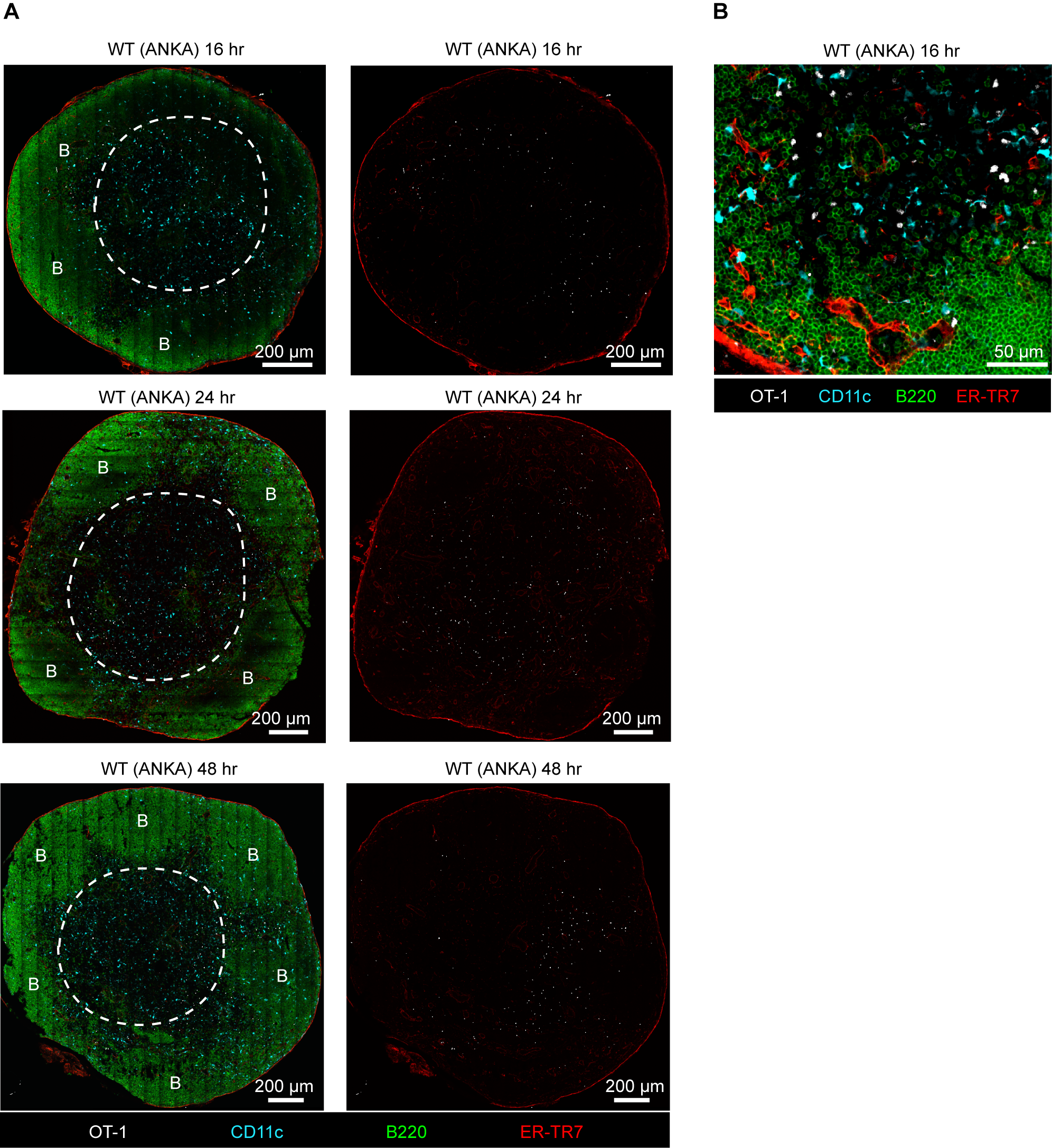

Supplement: S3 Fig — A. 2×10^6^ OT-1 cells were transferred to naïve mice 24 hours before ID inoculation with 1×10^5^ irradiated P. berghei ANKA (WT) sporozoites. Popliteal LNs were harvested at the indicated time points and confocal images of DLNs were prepared from 30 μm thick sections. White dotted line demarcates the cortex. B stands for B cell follicle. Representative images from 1 experiment with 2 mice per time point. B. Higher magnification of DLN section 16 hours after ID inoculation with 1×10^5^ irradiated P. berghei ANKA (WT) sporozoites. (TIF) [file ppat.1004637.s003.tif]

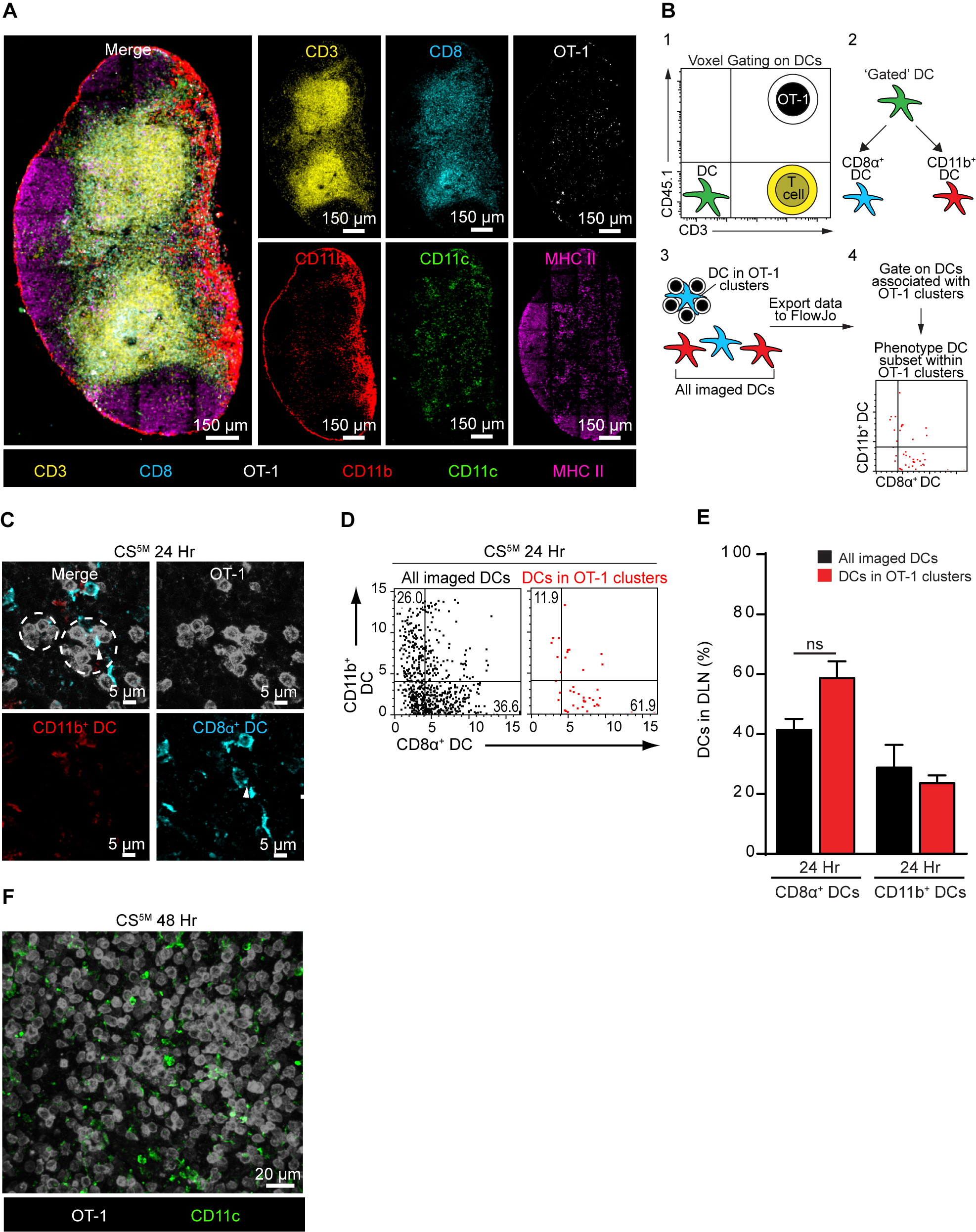

Supplement: S4 Fig — A. Confocal images of a representative LN section stained with a 6-color panel consisting of antibodies directed against CD3, CD8, CD45.1 (OT-1), CD11b, CD11c, and MHC II. B. Schema representing the theory behind histo-cytometry. Because CD8α is expressed on CD8+ T cells as well as a subset of DCs, we first had to generate a DC-specific channel by voxel gating on the CD11c+ CD3- CD45.1- population (Step 1). Once we obtained a ‘Gated DC’ channel, we examined the mean voxel intensities for the CD8 and CD11b channels within DCs (CD3- CD45.1- CD11c+) and generated new channels corresponding to CD8α+ and CD11b+ DC subsets (Step 2). To identify which DCs were presenting sporozoite antigens to the OT-1 cells, we generated a unique channel corresponding to OT-1 clusters using the imaging software Imaris (Step 3). We exported our imaging data to FlowJo and used the ‘OT-1 cluster’ channel to gate on DC populations within OT-1 clusters (Step 4). C-F. 2×10^6^ OT-1 (CD45.1+) cells were transferred to naïve mice 1 day before ID inoculation with 1×10^5^ irradiated P. berghei CS5M sporozoites. Popliteal LNs were harvested at the indicated time points, stained with the 6-color panel outlined in A, and imaged with a confocal microscope. C. IF image of OT-1 clusters taken 24 hours after ID inoculation of P. berghei CS5M sporozoites. White circles highlight representative OT-1 clusters. Arrowhead indicates OT-1 cluster-associated CD8α+ DC. D. Representative histo-cytometry scatter plots depicting the percentage of all imaged DCs (black dots) and DCs associated with OT-1 clusters (red dots) in DLNs 24 hours after ID inoculation of sporozoites. E. The percentages of cluster-associated CD8α+ or CD11b+ DCs (red bars) vs. all imaged DCs (black bars) were quantified from 2 independent experiments with 3 DLNs/time point (mean ± SEM). F. Representative image of robust proliferation of OT-1 cells 48 hours after ID inoculation of sporozoites. (TIF) [file ppat.1004637.s004.tif]

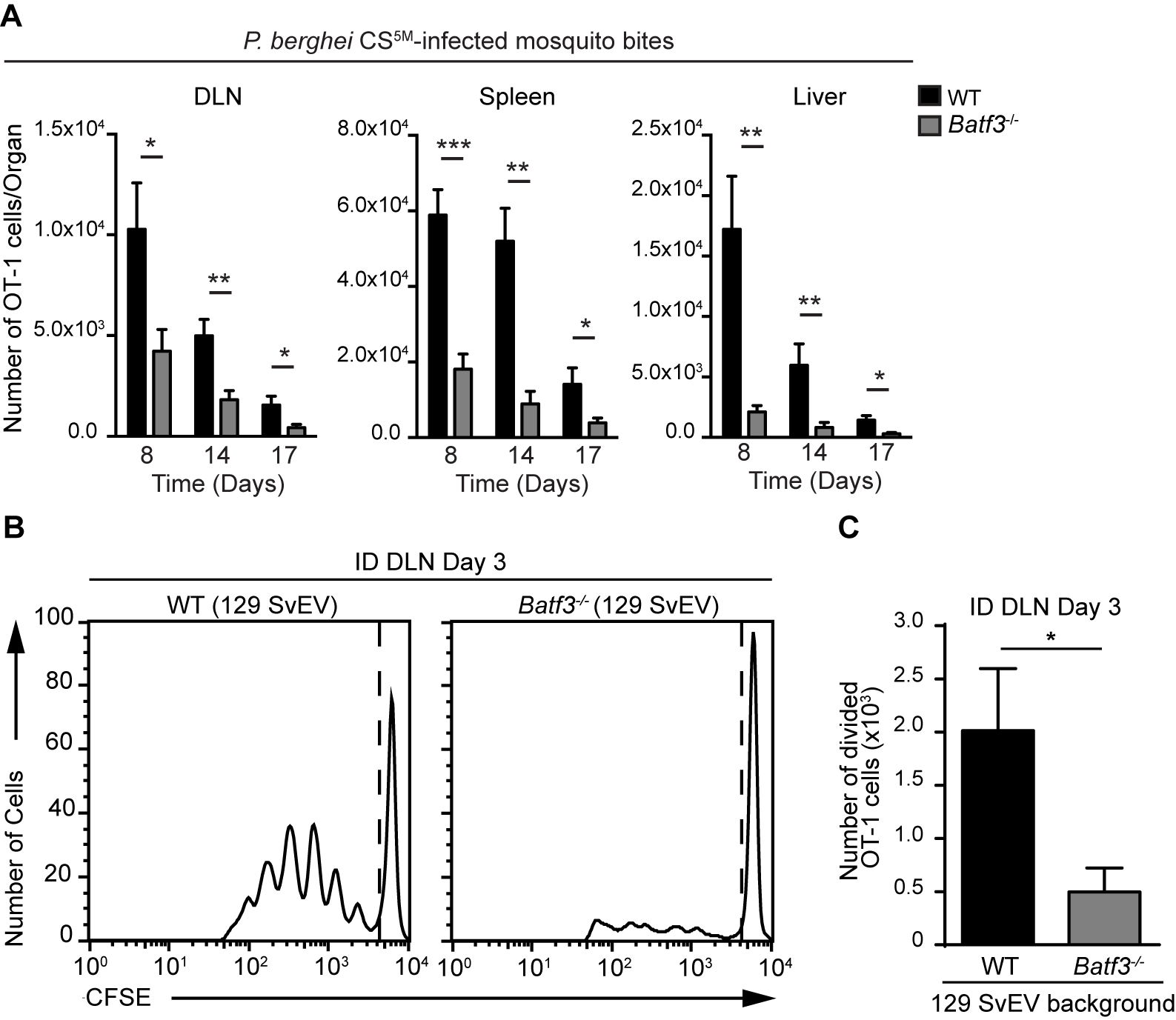

Supplement: S5 Fig — A. 0.5–1×10^4^ naive OT-1 cells were transferred to WT and Batf3 −/− mice 1 day before immunization via 20 irradiated P. berghei CS5M-infected mosquito bites. Responses were examined 8, 14, and 17 days post-inoculation. Number of OT-1 cells in the DLNs, spleen, and liver of immunized mice. Data are pooled from 2 similar experiments mean ± SEM; n = 9/group (day 8) and n = 6/group (day 14 and 17). B and C. To reduce the likelihood of OT-1 rejection by 129 SvEV recipient mice, we crossed OT-1 TCR transgenic mice on the C57BL/6 background to 129 SvEV mice. OT-1 cells were purified from 129 SvEV/C57BL/6 mice, CFSE-labeled, and adoptively transferred to Batf3 −/− mice on the 129 SvEV background or 129 SvEV control mice. Mice were immunized with 2×10^4^ irradiated P. berghei CS5M sporozoites 24 hours after transfer of 1×10^6^ OT-1 cells. DLNs were harvested 72 hours after sporozoite immunization and analyzed for CFSE dilution. B. Representative plots from WT and Batf3 −/− mice. C. Number of divided OT-1 cells pooled from 2 similar experiments (mean ± SEM; n = 6–7/group). (TIF) [file ppat.1004637.s005.tif]

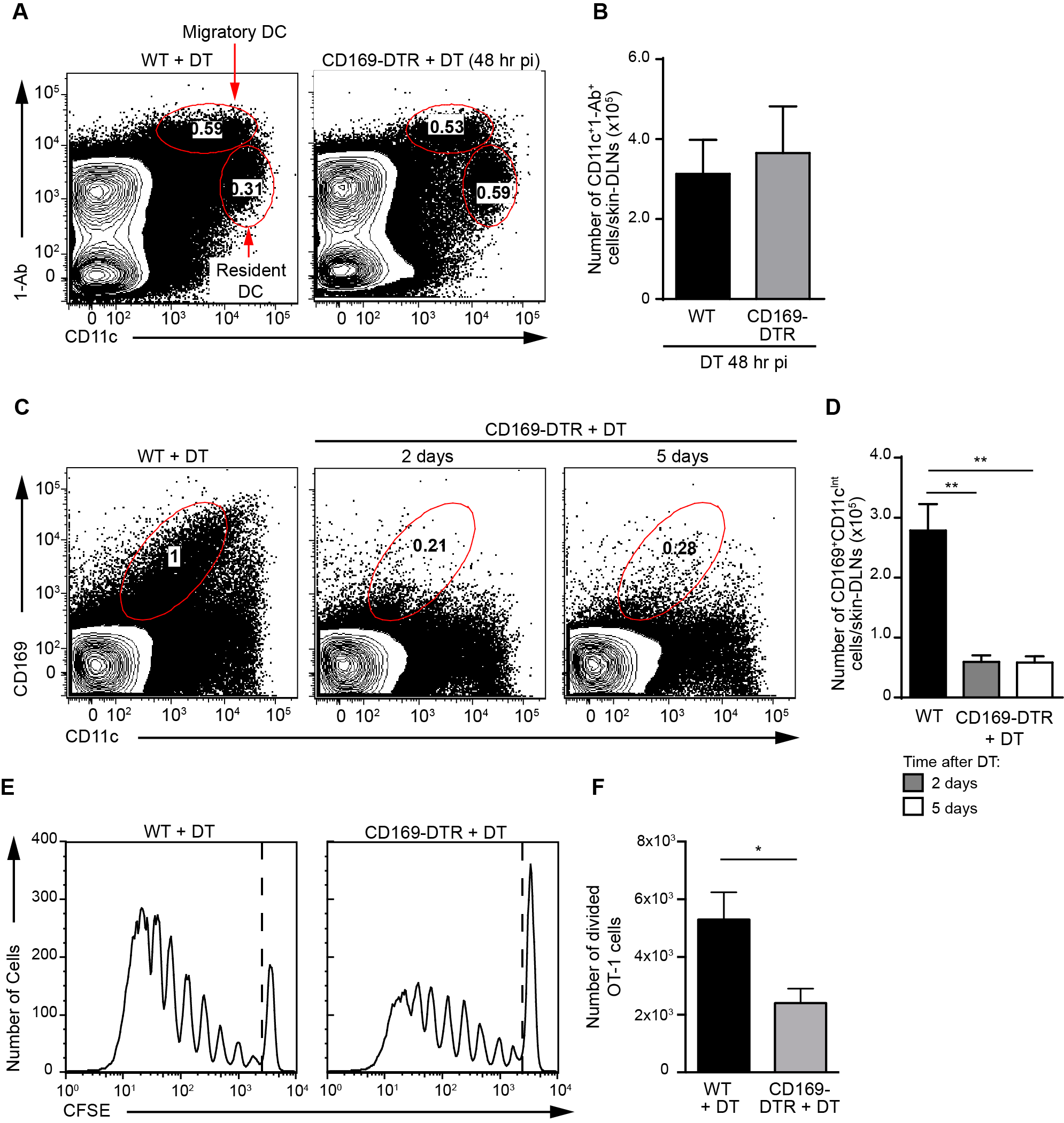

Supplement: S6 Fig — The skin-DLNs of WT and CD169-DTR mice were harvested 2 and 5 days after a single IP injection of 1 ug DT. Cells were gated on single, live cells. A. Percentage of migratory (CD11cInt 1-AbHi) and resident (CD11cHi 1-AbInt) DCs from the skin-DLNs from an individual mouse 48 hours after DT treatment. B. Number of conventional DCs from the skin-DLNs of individual WT and CD169-DTR mice; representative of 3–4 mice/group. C. Percentage of CD169+ cells in the skin-DLNs of WT and CD169-DTR mice 2 and 5 days after DT treatment. D. Number of CD169+ CD11cInt cells from the skin-DLNs of individual WT and CD169-DTR mice and representative of 3–4 mice/group. E and F. Mice were injected with 1 ug DT one day prior to adoptive transfer of 1×10^6^ purified, CFSE-labeled OT-1 cells to WT and CD169-DTR mice. 24 hours after cell transfer and 48 hours after DT treatment, mice were inoculated with 2×10^4^ irradiated P. berghei CS5M sporozoites ID. DLNs were harvested 72 hours after sporozoite immunization and analyzed for CFSE dilution. E. Representative CFSE dilution plots from WT and CD169-DTR mice treated with DT. F. Number of divided OT-1 cells (mean ± SEM; n = 3–5/group). The data presented here are from one independent experiment and are representative of 2–3 similar experiments. (TIF) [file ppat.1004637.s006.tif]
