# Supplementary material for: Lymph-Node Resident CD8α+ Dendritic Cells Capture Antigens from Migratory Malaria Sporozoites and Induce CD8+ T Cell Responses
Source: PLoS Pathog. 2015 Feb 6;11(2):e1004637. doi: 10.1371/journal.ppat.1004637 (PMC4450069; doi:10.1371/journal.ppat.1004637)
Supplement: S1 Methods — In addition, detailed protocols for immunofluorescence, flow cytometry, and parasite cloning are included in this section. (DOCX) [file ppat.1004637.s010.docx]

**S1 Methods**

**Mice**

5-8 week old female C57BL/6 female mice were purchased from NCI (Frederick, MD). *Batf3^-/-^* (B6.129S(C)-*Batf3^tm1Kmm^*/J strain #013755) and *Batf3^-/-^* (129S-*Batf3^tm1Kmm^*/J strain #013756) mice were purchased from Jackson Laboratories (Bar Harbor, ME). CD11c-EYFP mice were obtained from Bob Seder through a special contract with Taconic Laboratories and the National Institute of Allergy and Infectious Disease, Bethesda, MD. Langerin-DTR/EGFP mice were kindly provided by Miriam Merad of the Mount Sinai School of Medicine (New York, NY) with permission from Bernard Malissen of the CIML Parc Scientifique de Luminy Case (Marseille France). CD169-DTR mice were graciously provided by Tracy McGaha of the Georgia Regents University (Augusta, GA) with permission from Masato Tanaka of the Tokyo University of Pharmacy and Life Sciences (Tokyo, Japan) [1], [2]. Transgenic OT-1 mice expressing a TCR specific for the H-2K^b^-SIINFEKL ligand were kindly provided by David Sacks of the Laboratory of Parasitic Disease of the NIAID, Bethesda, MD. Ubiquitin promoter-tdTomato transgenic mice were generated by cloning the coding region of tdTomato (generous gift from Roger Tsien, University of California, San Diego, [3] down-stream of the human ubiquitin C promoter (kindly provided by Brian Schaefer, Uniformed Armed Services University, Bethesda, MD; [4]. Transgenic mice expressing GFP under the human ubiquitin C promoter were purchased from Jackson Laboratories (Bar Harbor, ME). Founder lines were established through backcrossing with C57BL/6 mice and a single founder line was selected based on high and uniform levels of tdTomato or GFP fluorescence in hematopoietic cells.

**Whole ear mount immunofluorescence**

Ears were excised from mice, split into dorsal and ventral halves, and fixed for 12 hours at 4°C with PLP buffer (0.05 M phosphate buffer containing 0.1 M L-lysine [pH 7.4], 2 mg/ml NaIO_4_, and 10 mg/ml paraformaldehyde). Following fixation, samples were incubated for 12 hours at 4°C in blocking solution (0.5% bovine serum albumin and 0.3% Triton-X-100 in PBS). Samples were then stained with rat anti-langerin antibody (clone eBio31) in blocking solutions for 2 days at 4°C with gentle shaking, followed by an appropriate secondary antibody for 12 hours at 4°C with gentle shaking. Stained skin samples were mounted dermal side down in Fluoromount G (eBioscience), and acquired on a 710 confocal microscope (Carl Zeiss Microimaging). Images were analyzed with Imaris software (Bitplane).

**DT Depletion in DTR mice**

Langerin-DTR/EGFP heterozygous mice, CD169-DTR heterozygous mice, and WT mice received a single IP injection of 1 ug DT (Sigma) 2 days before sporozoite immunization.

***Ex vivo* Stimulation**

EL4.IL-2 cells (ATCC) were pulsed with SIINFEKL peptide (10 μg/ml) and control EL4 cells were incubated without peptide at 37°C for 1 hour. Peptide-coated or control target cells were washed and added to lymphocytes harvested from different tissues of sporozoite-injected mice. The cells were stimulated for 4 hours at 37°C in the presence of Golgi Stop (BD Biosciences) and Golgi Plug (BD Biosciences) followed by intracellular staining using the BD Cytofix/Cytoperm solution kit (BD Biosciences).

**Isolation of APC subsets in DLNs**

DLNs were harvested, minced into small pieces, and incubated with 1 mg/ml collagenase D (Sigma) and 15 ug/ml DNase (Roche) for 30 minutes at 37˚C. Single cell suspensions were prepared from the DLNs by grinding the digested fragments between the rough sides of two microscope slides. Cells were then stained with the viability dye LIVE/DEAD Fixable Aqua Dead Cell Stain (Invitrogen) and stained with the following antibodies: CD8, CD11b, CD11c, CD169, CD207, DEC-205, and MHC-II. Intracellular staining using the BD Cytofix/Cytoperm solution kit (BD Biosciences) was performed to detect CD207 (langerin).

**Preparation of Fab monomer fragments**

Fab monomers were generated from the mouse monoclonal IgG antibody recognizing the repeat region of *P. berghei* circumsporozoite protein (3D11) by digesting 2 mg of 3D11 in a solution of 43.9 mg cysteine-HCl in 10 ml of mouse IgG digestion buffer for 4 hours at 37˚C with gentle shaking as outlined in the instructions for the Pierce Mouse IgG_1_ Fab and F(ab’)_2_ Preparation Kit (Thermo Scientific, Rockford, IL). Fab monomers were separated from undigested monoclonal antibody and Fc fragments by passing the digestion reaction over a Protein A column two times. SDS-PAGE was used to assess the purity of the Fab monomer preparation. An anti-*P. knowlesi* CS (2G3) mAb was used as an isotype control in the antibody-mediated sporozoite immobilization studies.

**Construction of *P. berghei CS^5MΔN^* parasites**

*P. berghei* CS^5MΔN^ parasites were created by transfection of *P. berghei* ANKA with linearized pR-CSRepΔN5M plasmid. The pR-CSRepΔN5M plasmid was generated by digesting the pCSRep5M plasmid with the restriction enzymes EagI and PacI. The EagI-PacI digestion product was ligated into the pR-CSRepΔN plasmid containing the N-terminal deletion of the *CSP* locus and a drug selection cassette [5]. Mutant parasites were selected by pyrimethamine and cloned by limiting dilution. Clones were sequenced and the following primers were used to screen for the N-terminal deletion of *P. berghei* CS: CS129F: 5’-AGAGAAGATCAGGGCTTGTT-3’ and CS1484R: 5’- GTTACGTTACATTGAGACCA-3’, yielding an expected product of 1.3 Kb with genomic DNA from *P. berghei* CS^5M^ parasites and a 1.1 Kb product with genomic DNA from *P. berghei* CS^5MΔN^ clones. To verify the presence of the SIINFEKL epitope in *CSP* and stable genomic integration, PCR was performed with the following primers: DNOVA 2F 5’-ATGACGATTCTATCATCAATTTCG-3’ and CS4 5’-CGAAATAAGTTACTATTCGTGCCC-3’.

**Immunofluorescence and histo-cytometry**

DLNs were harvested and fixed with PLP buffer (0.05 M phosphate buffer containing 0.1 M L-lysine [pH 7.4], 2 mg/ml NaIO_4_, and 10 mg/ml paraformaldehyde) for 12 hours. Following fixation, DLNs were incubated in 30% sucrose for 6 hours before embedding in OCT compound (Tissue-Tek). 25-45 μm sections were cut on a CM3050S cryostat (Leica) and adhered to Super Frost Plus Gold slides (Electron Microscopy Services). Frozen sections were permeabilized and blocked for 1-2 hours in PBS containing 0.3% Triton X-100 (Sigma), 1% normal mouse serum, 1% bovine serum albumin, and 10% normal goat serum. Endogenous biotin, biotin receptors, and avidin binding sites were blocked using an Avidin/Biotin blocking kit (Vector Laboratories) before staining with biotin-conjugated antibodies. The Mouse-on-Mouse (M.O.M) basic kit (Vector Laboratories) was used to eliminate non-specific background in applications using the mouse mAb directed against *P. berghei* CS (3D11). Sections were stained with directly conjugated antibodies or appropriate primary and secondary antibodies for a minimum of 5 hours at RT or 12 hours at 4˚C in a humidity chamber in the dark. Stained slides were mounted with Fluoromount G (eBioscience) and sealed with a glass coverslip. Each section was visually inspected by epifluorescent light microscopy and several representative sections from different LNs were acquired using a 710 confocal microscope (Carl Zeiss Microimaging), objectives with 40X magnification (NA 1.1) or 63X magnification (NA 1.2), or a SP8 confocal microscope (Leica), objectives with 40X magnification (NA 1.3) or 63X magnification (NA 1.4). For histo-cytometric analysis of OT-1 cluster-associated DCs, we developed a 6-color panel consisting of the following fluorophores: Brilliant Violet 421, Alexa Fluor 488, Brilliant Violet 510, Alexa Fluor 568, Alexa Fluor 647, and Alexa Fluor 700. Fluorophore emission was collected on separate detectors with sequential laser excitation used to minimize spectral spillover. The Channel Dye Separation module within the LAS AF software (Leica) was then used to correct for any residual spillover. In order to identify the DC subset(s) presenting antigen to OT-1 cells, we serially sectioned and stained whole DLNs from mice that received CD45.1^+^ OT-1 cells one day prior to ID injection of 1x10^5^ irradiated *P. berghei* CS^5M^ sporozoites. For each DLN, 10 to 16 z-stacks of OT-1 clusters were taken at a voxel density of 1024x1024 and 1 µm z step using a SP8 confocal microscope equipped with a 63X (1.4A) objective. Individual sections from independent DLNs were analyzed as a batch to ensure uniform analysis. Threshold identification, voxel gating, surface creation, masking, and signal segmentation was performed as previously described [6]. Channel statistics for all surfaces were exported into Excel (Microsoft) and converted to a csv file for direct visualization in FlowJo v10 (Treestar). Mean voxel intensities for the CD8 and CD11b channels gated within DCs (CD3^-^ CD45.1^-^ CD11c^+^ voxels) were plotted on a linear scale. To identify DCs within the OT-1 clusters, we first created a surface for OT-1 clusters with a volume greater than 350 µm^3^ using the Surface Creation Module in Imaris (Bitplane). We next masked this surface and set all values within the surface (OT-1 clusters) to 100 and all values outside of the surface (non-clustered OT-1 cells) to zero. This process generated a new channel (OT-1 clusters) that could be used to gate on DC populations within OT-1 clusters. For the 8 and 16 hour time points, 4 DLNs from 3 independent experiments were analyzed. For the 24 and 48 hour time points, 3 DLNs from 2 independent experiments were analyzed.

**Multi-photon intravital imaging**

Mice were anesthetized with isoflurane (Baxter; 2.5% for induction, 1-1.5% for maintenance, vaporized in an 80:20 mixture of O_2_ and air). 1x10^5^ *P. berghei* CS^5M^ GFP sporozoites were injected ID into the footpads of mice and popliteal LNs were exposed and imaged using a protocol modified from [7]. The imaging system was composed of a Zeiss 710 microscope equipped with a Chameleon laser (Coherent) and a femtosecond fiber laser (PolarOnyx, 1050 nm) as well as a 20X water dipping lens (NA 1.0, Zeiss). Dynamic imaging experiments were performed in an environmental chamber and the surgically-exposed LN was kept at 30˚C with warmed PBS. A z stack of 60 μm with a 3 μm step size was acquired every 40 sec. Lymph was visualized by subcutaneous injection of 1ul Qdot 705 (Invitrogen) in PBS. Raw imaging data were processed and analyzed with Imaris software (Bitplane).

**Antibodies**

All antibodies were purchased from eBioscience unless stated otherwise. The following ﬂuorochrome-conjugated monoclonal antibodies were used: anti-B220 (clone RA3-682), anti-CD3 (clone 17A2), anti-CD8 (clone 53-6.7), anti-CD11b (clone 5C6, AbD Serotec), anti-CD11b (clone M1/70), anti-CD11c (clone N418), anti-CD45.1 (clone A20), anti-CD69 (clone H1.2F3), anti-CD103 (clone 2E7), anti-CD169 (clone 3D6.112, AbD Serotec), anti-DEC205 (clone NLDC-145, Miltenyi), anti-CD207 (clone eBioL31), anti-ERTR-7 (Acris), anti-IFN-γ (clone XMG1.2), anti-Lyve-1 polyclonal rabbit (Acris), anti-MHC II (M5/114.15.2), and Vα2 (clone B20.1). Unconjugated primary antibodies were stained with Alexa Fluor-conjugated secondary antibodies (Invitrogen), streptavidin Alexa Fluor 350 conjugate (Invitrogen), or streptavidin Brilliant Violet 510 (BioLegend). Flow cytometric data was collected on a FACSCalibur (Becton Dickinson) or an LSRII flow cytometer (Becton Dickinson).

**S1 Methods References**

1. Miyake Y, Asano K, Kaise H, Uemura M, Nakayama M, et al. (2007) Critical role of macrophages in the marginal zone in the suppression of immune responses to apoptotic cell-associated antigens. J Clin Invest 117: 2268-2278.

2. Saito M, Iwawaki T, Taya C, Yonekawa H, Noda M, et al. (2001) Diphtheria toxin receptor-mediated conditional and targeted cell ablation in transgenic mice. Nat Biotechnol 19: 746-750.

3. Shaner NC, Campbell RE, Steinbach PA, Giepmans BN, Palmer AE, et al. (2004) Improved monomeric red, orange and yellow fluorescent proteins derived from Discosoma sp. red fluorescent protein. Nat Biotechnol 22: 1567-1572.

4. Schaefer BC, Schaefer ML, Kappler JW, Marrack P, Kedl RM (2001) Observation of antigen-dependent CD8+ T-cell/ dendritic cell interactions in vivo. Cell Immunol 214: 110-122.

5. Coppi A, Natarajan R, Pradel G, Bennett BL, James ER, et al. (2011) The malaria circumsporozoite protein has two functional domains, each with distinct roles as sporozoites journey from mosquito to mammalian host. J Exp Med 208: 341-356.

6. Gerner MY, Kastenmuller W, Ifrim I, Kabat J, Germain RN (2012) Histo-cytometry: a method for highly multiplex quantitative tissue imaging analysis applied to dendritic cell subset microanatomy in lymph nodes. Immunity 37: 364-376.

7. Bajenoff M, Egen JG, Koo LY, Laugier JP, Brau F, et al. (2006) Stromal cell networks regulate lymphocyte entry, migration, and territoriality in lymph nodes. Immunity 25: 989-1001.
